# Reveal cell type-specific regulatory elements and their characterized histone code classes via a hidden Markov model

**DOI:** 10.1186/s12864-018-5274-9

**Published:** 2018-12-31

**Authors:** Can Wang, Shihua Zhang

**Affiliations:** 1 Academy of Mathematics and Systems Science, Chinese Academy of Sciences, Beijing, 100190 China; 2 School of Mathematical Sciences, University of Chinese Academy of Sciences, Beijing, 100049 China; 3 Center for Excel-lence in Animal Evolution and Genetics, Chinese Academy of Sciences, Kunming, 650223 China

**Keywords:** Epigenomics, Cell type-specific regulatory elements, Hidden Markov model, Histone modification

## Abstract

**Background:**

With the maturity of next generation sequencing technology, a huge amount of epigenomic data have been generated by several large consortia in the last decade. These plenty resources leave us the opportunity about sufficiently utilizing those data to explore biological problems.

**Results:**

Here we developed an integrative and comparative method, CsreHMM, which is based on a hidden Markov model, to systematically reveal cell type-specific regulatory elements (CSREs) along the whole genome, and simultaneously recognize the histone codes (mark combinations) charactering them. This method also reveals the subclasses of CSREs and explicitly label those shared by a few cell types. We applied this method to a data set of 9 cell types and 9 chromatin marks to demonstrate its effectiveness and found that the revealed CSREs relates to different kinds of functional regulatory regions significantly. Their proximal genes have consistent expression and are likely to participate in cell type-specific biological functions.

**Conclusions:**

These results suggest CsreHMM has the potential to help understand cell identity and the diverse mechanisms of gene regulation.

**Electronic supplementary material:**

The online version of this article (10.1186/s12864-018-5274-9) contains supplementary material, which is available to authorized users.

## Background

With the rapid development of sequencing technologies [1], a myriad of epigenomic data have been generated by both large consortia such as ENCODE [2], modENCODE [3], Roadmap Epigenomics Project [4], and many independent laboratories. Those data involve histone modifications, chromatin openness, DNA methylation, nucleosome positioning and so on. Among them, histone modifications have over 100 types, and the combinatorial presence of them are closely related to distinct patterns of gene regulation. For example, H3K4me1 and H3K27ac were successfully used to identify genome-wide enhancers. In contrast, combination of H3K4me1 and H3K27me3 was a well-studied marker of poised enhancers [5]. With the plenty of epigenomic data available, there is a challenge in computational biology to decode the abundant information hidden behind the functional regulatory elements.

To this end, dozens of computational tools have been developed in the past decade [6–15]. ChromHMM [6] is a typical one used by big consortia to generate genome-wide chromatin annotations for diverse cell types based on ChIP-seq peaks of chromatin modifications, transcription factors and DNaseI hypersensitive sites. It utilizes a multivariate hidden Markov model with independent Bernoulli distribution to learn the underlying chromatin states. The algorithm converts raw signals in 200-bp non-overlapping bins into binary values based on the Poisson distribution and then concatenates the epigenomes of multiple cell types to jointly learn the segmentation. Other methods extended such an algorithm from different views recently. For example, EpiCSeg [7] and GenoSTAN [8] adapted modeling of emission probability to fit raw count or signal for gaining more information. TreeHMM [9], hiHMM [10] and IDEAS [11] applied more sophisticated hidden structures to reveal relationship between cell types or species. Spectacle [12] leveraged spectral learning to explicitly model mark combinations and accelerate training process. BdHMM [13] and dsHMM [14] took direction into account to better annotate gene structure on both strands of DNA. GBR-Segway [15] integrated Hi-C data with histone combinations to better annotate the genome.

Although, these methods facilitated the determination and characterization of various chromatin states for a cell type, they do not explore differences between epigenomes of cell types directly, which could provide novel information of cell type-specific biological functions and cell identity [16]. To directly identify cell type-specific regulatory elements (CSREs) by comparing epigenomes, Chen et al. [17] proposed a differential Chromatin Modification Analysis (dCMA) strategy, and defined CSREs for nine cell lines. Wang and Zhang [18] adapted this method to determine CSREs across 127 cell types and tissues for a comprehensive characterization of the CSREs and their funcitons. Their analyses found that epigenomic modifications function in cell type-specific manners and CSREs show significant, cell-type-specific biological relevance and tend to be regulatory elements. However, dCMA only locates CSREs for each cell type, but does not directly characterize their underlying specific histone codes. Besides, the CSREs shared by multiple cell types reveal important common functions among them, which were found via overlap analysis for a given group of cell types, but could not be done automatically by dCMA.

To this end, we developed a hidden Markov model to systematically identify CSREs (CsreHMM). Compared to dCMA, this method can additionally learn the subclasses of CSREs and their characterized histone codes directly, which is necessary to explicitly illustrate the diverse functions of CSREs. Besides, CsreHMM could naturally identify groups of cell types which tend to share common CSREs, revealing the common functions among those cell types. We first applied it to a data set of 9 cell types and 9 chromatin marks to demonstrate its effectiveness. The identified CSREs show distinct tendency to known functional regulatory regions. Their proximal genes have consistent expression and are likely to participate in cell type-specific biological functions. These results suggest the HMM model can not only determine significant functionally relevant CSREs, but also reveal CSRE-related specific histone codes which have the potential to help understand the gene regulation and cell identity.

## Methods

### Data

We downloaded the ChIP-seq data of 9 chromatin marks as well as a whole-cell extract (WCE) control across 9 cell types [19]. The cell types consist of human embryonic stem cells (H1), chronic myelogenous leukemia (K562), lymphoblastoid (GM12878), hepatocellular carcinoma (HepG2), human umbilical vein endothelial cells (HUVEC), human skeletal muscle cells and myoblasts (HSMM), normal human lung fibroblasts (NHLF), normal human epidermal keratinocytes (NHEK), and human mammary epithelial cells (HMEC). The nine chromatin marks include CTCF, H3K27me3, H3K36me3, H4K20me1, H3K4me1/2/3, H3K27ac, and H3K9ac. Besides, a whole-cell extract (WCE) was also sequenced as the control for each cell type. From GSE26386, we downloaded the reads that have been aligned to hg18 by MAQ (http://maq.sourceforge.net/maq-man.shtml). For each pair of cell type and mark, replicates were merged and peaks were called. Specifically, the whole genome was divided into 200-bp non-overlapping bins. Each read was extended to 200-bp from 5′ end to 3′ direction and then was assigned to a bin by its midpoint. The peaks were called based on a Poisson background model with *λ* equaling the average read counts across all bins with a threshold of 10^− 4^.

### Input for HMM

**Fig. 1:**
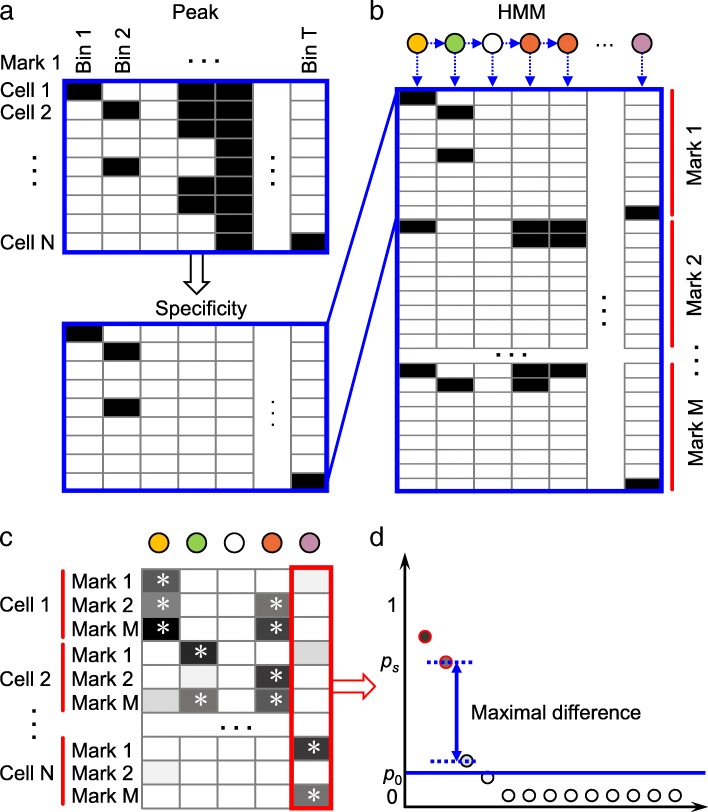
Illustration of the workflow of CsreHMM. **a** Transform the binary peak matrix to the specificity matrix for each mark. **b** Apply HMM to the feature matrix formed by stacking all specificity matrices of all marks to learn specific states. **c** The emission matrix of trained HMM model. For each state, the specific cell-mark combination is marked by ‘*’. Only state with at least one specific cell-mark combination was defined as a specific state (marked by a colored circle). **d** A specific cell-mark combination is defined as the one with emission probability above both *p*_0_ and *p*_*s*_ (explained in Methods)

For each mark *m* (one of CTCF, histone marks and WCE), we have a *N* by *T* peak matrix *P*^(*m*)^ with rows standing for *N* cell types and columns indicating *T* bins along the whole genome (Fig. 1a). Each element in *P*^(*m*)^ has the following meaning:

$$ {P}_{ij}^{(m)}=\left\{\begin{array}{cc}1,& cell\ type\ i\  has\ a\  peak\ of\ mark\ m\  on\  bin\ j\\ {}0,& otherwise\end{array}\right. $$

To extract specificity information, we transformed the peak matrix *P*^(*m*)^ to a specificity matrix *S*^(*m*)^. In detail, for each bin *j*, if there were no more than *s* cell types (*s* = 2 for data used here) that have signal on that bin, then we considered that those cell types were specific, and kept ${S}_{.j}^{(m)}={P}_{.j}^{(m)}$, otherwise set ${S}_{.j}^{(m)}=0.$ Thus, *S*^(*m*)^ has the following format:

$$ {S}_{ij}^{(m)}=\left\{\begin{array}{cc}1,& cell\ type\ i\  is\ specific\  on\  bin\ j\  according\ to\ mark\ m\\ {}0,& otherwise\end{array}\right. $$

To catch the combinatorial information of multiple marks on each bin, we stacked *S*^(*m*)^ for all *M* marks to form a *MN* by *T* matrix *O* = (*S*^(1)^; *S*^(2)^; …; *S*^(*M*)^) (Fig. 1b). Each row of *O* stands for a cell-mark combination, indicating whether the cell is specific according to that mark. Then we treated the columns of matrix *O* as observations and trained a multivariate HMM model to reveal the hidden states behind them.

### The HMM model

As the number of all possible observations are up to $$ {\left(\sum \limits_{i=1}^s\left(\begin{array}{c}i\\ {}N\end{array}\right)\right)}^M $$ (~3.4 × 10^16^ for the data used here), it is not practical to directly model the probability for each possible observation by one parameter. Instead, we used a Bernoulli random variable to model the probability of presence of a specific cell-mark combination, and a product of those *M* × *N* probabilities to model the total observation vector. Specifically, we assume there are *K* hidden states. For each pair of the *K* states, and *R* cell-mark combinations, there is an emission parameter *p*_*k*, *r*_ denoting the probability of observing the specific cell-mark combination *r* under state *k*. The *T* bins are from *C* chromosomes, each with *T*_*c*_ bins. For each chromosome *c*, let (*c*, *t*) denote the *t* th bin of *c*. The hidden state of bin (*c*, *t*) is denoted as *s*_(*c*, *t*)_. Let *a*_*i*, *j*_ denote the probability of transitioning from state *i* to *j*. And let *π*_*i*_ denote the probability that the state of the first interval on each chromosome is *i*. Then the likelihood of all observations can be formulated as:

$$ P\left(O|\pi, a,p\right)=\prod \limits_{c=1}^C\sum \limits_{s_{\left(c,1\right)},\dots, {s}_{\left(c,{T}_c\right)}}{\pi}_{s_{\left(c,1\right)}}\left(\prod \limits_{t=2}^{T_c}{a}_{s_{\left(c,t-1\right)},{s}_{\left(c,t\right)}}\right)\prod \limits_{t=1}^{T_c}\prod \limits_{r=1}^R{p}_{s_{\left(c,t\right)},r}^{O_{r,\left(c,t\right)}}{\left(1-{p}_{s_{\left(c,t\right)},r}\right)}^{1-{O}_{r,\left(c,t\right)}} $$

As there are hidden variables, we maximize the likelihood using the incremental expectation-maximization algorithm, which is a variant of Baum-Welch algorithm for accelerating the training process with multiple observations [20].

There are many ways to initialize the parameters of HMM model in literature. For example, several studies used random initializations [21]. Several studies used *k*-means clustering to get an initial segmentation and estimate parameters [8]. And several studies used entropy-based method to segment the genome and estimate parameters [20]. Among them, the entropy-based method gives similar initializations for models with different number of states. Hence, models with such initialization would be more comparable across different number of states. Thus, we utilized the entropy-based method to initialize our HMM model.

### Determination of specific states

To determine which states are specific to which cell types, we utilized the emission probabilities (Fig. 1c and d; and Additional file 1: Figure S1). For each state, we sorted the emission probabilities of all cell-mark combination decreasingly and found the maximal difference. The probability above it was denoted as *p*_*s*_. To remove small probabilities, we also set a threshold *p*_0_ (0.3 was used in this study). Only the cell-mark combination with probability passing both *p*_*s*_ and *p*_0_ was defined as a specific one. Then the specific state was defined as one with at least one specific cell-mark combination. The name of each specific state was based on its corresponding cell types. A region consisting of consecutive bins covered by a specific state was defined as a cell type-specific regulatory elements (CSRE).

### Model selection

We trained models with number of states from 5 to 70, increased by every 5 states. We found that each model converged during training procedure within 300 iterations, which means we got a local maximal for the log likelihood. We calculated the BIC and AIC scores as BIC = ln(#bins) ×  # parameters − 2 ln(likelihood) and AIC = 2 ×  # parameters − 2 ln(likelihood), respectively, where #parameters = (#states − 1) +  # states × (#states − 1) +  # states ×  # cells ×  # marks. We observed that both BIC and AIC scores are monotonically decreasing as number of states is increasing (Additional file 1: Figure S2). Even model of 70 states may not be a minimal. However, for 70-state model, there are lots of similar cell-type-specific states, which cannot be distinguished well with emission probabilities (Additional file 1: Figure S3). Thus, neither BIC nor AIC is a proper criterion for selecting a proper model. Finally, we selected the 30-state model to do downstream analyses. One reason is that the log-likelihood is increasing smoothly from 30- to 70-state models. Another important reason comes to the fact that the 30-state model begins to harbor a specific state marked by H3K36me3.

We also investigated the robustness of specific states to models with different initializations or different numbers of states. For each state *s* in the 30-state model, we defined its recovery score *V*_*s*, *H*_ in another model *H* as:

$$ {V}_{s,H}=\underset{s^{\prime}\in H}{\max } cor\left({p}_s,{p}_{s^{\prime }}\right), $$

where *p*_*s*_ = (*p*_*s*, 1_, *p*_*s*, 2_, …, *p*_*s*, *R*_), and *s*^′^ is a state in model *H*. We trained ten 30-state models with random initializations. All of them converged within 500 iterations. We found that the specific states have significantly higher recovery scores than non-specific ones (Additional file 1: Figure S4A and B) which demonstrated the robustness of our results. We also trained models with different numbers as aforementioned. Models with number of states larger than 30 preserve all states in the 30-state model, and hence use additional states to learn other patterns (Additional file 1: Figure S5).

### Mapping CSREs to various genomic features

We examined the potential functional relevance of CSREs by mapping them to known genomic features. We leveraged RefSeq annotation to build a TxDb object in Bioconductor on December 12, 2016 and extracted genomic features therein [22, 23]. Each transcript named with a prefix of “NM” by RefSeq was regarded as a gene here. Beyond that, we defined six genomic features: promoter, 5’UTR, 3’UTR, exon, intron and intergenic region. Promoters were defined as regions within 2000 bp of a transcription start site (TSS) and intergenic regions were composed of base pairs in none of the other five features. We assigned each CSRE to one of its overlapping features according to the order: promoter > 5’UTR > 3’UTR > exon > intron > intergenic region.

CSRE proximal genes were defined with a stringent criterion. Only genes with a consecutive 3 kb region within their promoters and bodies covered by CSREs from a specific state are defined as CSRE proximal genes for that state.

### Gene expression and specificity

Microarray data were downloaded for all 9 cell types from GSE26386. First, we used RMA to process the raw CEL files. The replicate expression values from the same cell types were then averaged. Next, the expression values of probe sets were averaged according to their corresponding RefSeqs. Finally, the average values were quantile normalized across 9 cell types and used as the expressions.

For each gene, we computed its *z*-scores of expressions across cell types and defined them as gene specificity scores. High positive (or low negative) specificity score indicates specific high (or low) expression for a gene. Difference of gene specificity scores for groups was tested by two-sample Wilcoxon test.

### GO enrichment analysis

We explored the biological functions of CSRE proximal genes by GO enrichment analysis. Each set of concerned genes were mapped to GO terms by org.Hs.eg.db and GO.db Bioconductor packages. Fisher’s exact test was used to get the *P*-values, which were then corrected by Benjamini-Hochberg method for each cell type. Only GO terms with 5 to 500 genes were kept.

### Cell type-specific DNase and EP300 peaks

We obtained the DNase and EP300 peaks from ENCODE by AnnotationHub and then transformed them from hg19 to hg18 version by the liftOver function of rtracklayer. DNase and EP300 peaks were available for 9 and 4 cell types, respectively. Cell type-specific DNase or EP300 peaks of a cell type were defined as part of original peaks that were not covered by peaks from any other cell types. To examine the relationship between CSREs from each specific state, and specific DNase or EP300 peaks in the corresponding cell types, we calculated the overlapping number of them. We randomly sampled 1000 sets of false CSREs for each specific state with length and chromosome reserved and calculated the overlapping number as genome-wide background observations. Then, one-sample Wilcoxon test was used to evaluate the statistical significance of the real number of overlapped ones.

### Applying CsreHMM to the roadmap Epigenomics dataset

We downloaded the signals of epigenomic modification tracks [−log_10_(*P*-value)] for five histone marks of 127 tissues and cell types (Additional file 2: Table S1) generated by the Roadmap Epigenomics Consortium at http://egg2.wustl.edu/roadmap/data/. The -log_10_(*P*-value) was generated by MACS2. We averaged the signal on each 200-bp non-overlapping bin and binarize it by threshold 2, which is recommended by the Roadmap Epigenomics Consortium. The histone marks consist of H3K4me1, H3K4me3, H3K36me3, H3K27me3 and H3K9me3, which relate to regulatory elements, promoters, transcribed chromatin, Polycomb-repressed regions and heterochromatin, respectively.

We trained a 30-state model with *s* = 5 for the 127 cell types or tissues and a 20-state model with *s* = 2 for 9 cell types of group “HSC & B cell”. The emission probabilities were analyzed and GO enrichment analysis was conducted for proximal genes of each state as aforementioned.

## Results

### Diversity of specific states

**Fig. 2:**
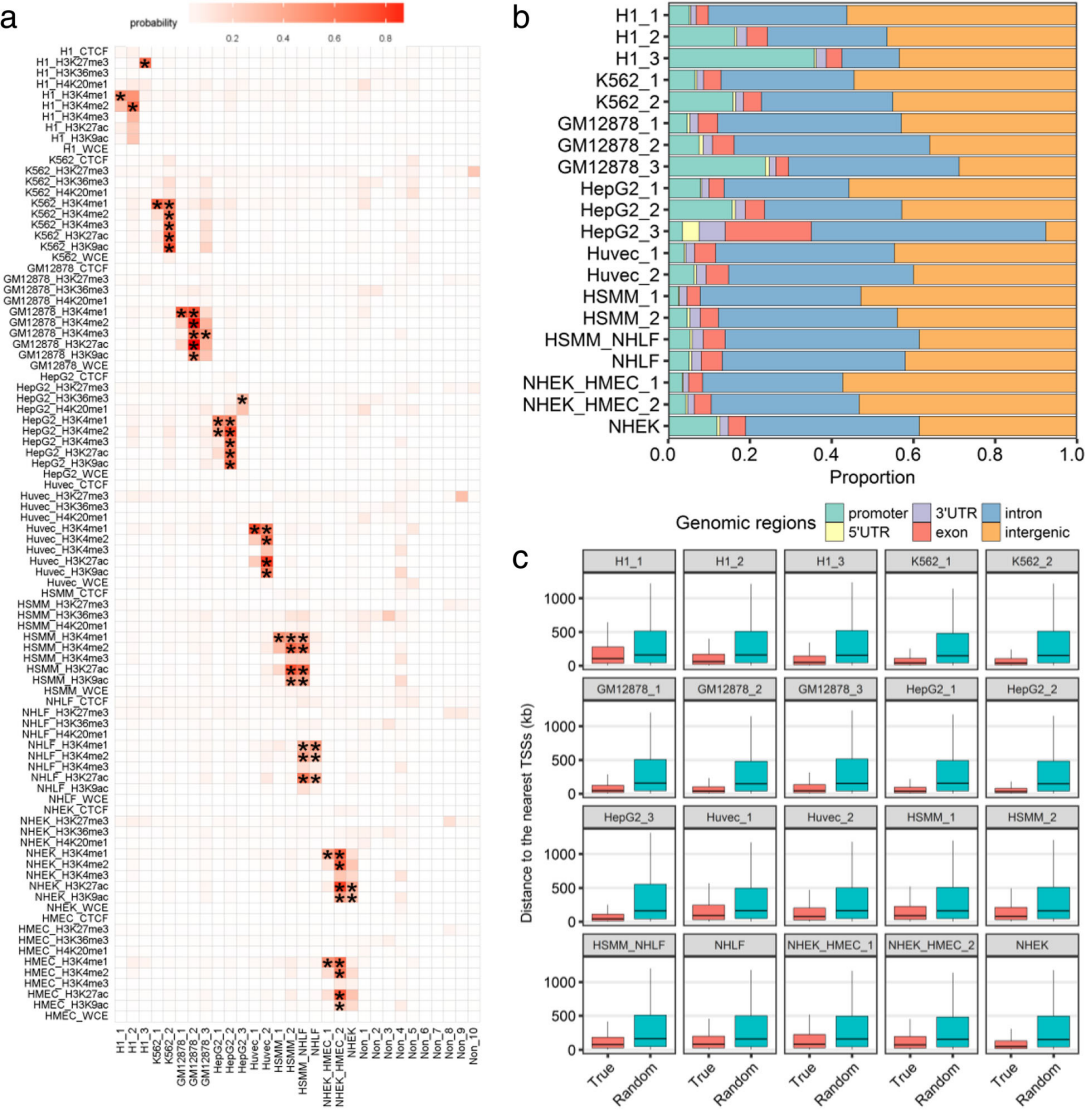
Characteristics of specific states. **a** Emission probabilities of 30 states. Specific cell-mark combinations are marked by ‘*’. Non-specific states are named with prefix “Non”. **b** Distribution of CSREs in six categories of genomic regions for each specific state. **c** Box plot of the distances between the intergenic CSREs and their nearest TSSs, compared with those of randomly generated ones. For each intergenic CSRE, the random one is an arbitrarily selected genomic element from the same chromosome with the same length. Then, the distances between the random regions to their nearest TSS were computed. Statistical significance between ‘True’ and ‘Random’ is calculated by two-sample Wilcoxon test with *P* < 2.2e–16 for all specific states

We trained a HMM model of 30 states on the data set of 9 cell types and 10 marks. Twenty of those are identified as specific ones, which covers 20% of the whole genome (Fig. 2a, Additional file 3). Even though the specific regions are only a small part of the genome, this model automatically suggests 2/3 states to describe them, which shows its effectiveness. WCE (having signals in regions with copy number variations (CNVs) or repeats [19]) is not specific for any cell type in any of the 20 specific states, indicating that all the specific states are indeed caused by differences of epigenomic marks, other than CNVs or repeats. Moreover, CTCF is also not specific for any cell type. This is consistent with previous studies which have shown that CTCF localization is largely invariant across different cell types [24]. Some histone modifications are only specific in one of the 9 cell types, such as H3K27me3 for H1 and H3K36me3 for HepG2, indicating those cell types own their distinct specific histone modifications. H3K4me1 is the unique specific mark for 6 states, indicating that it is the most commonly specific mark. There are also 9 states harbor no less than 3 active marks, confirming that there are combinatorial specific histone modifications. Interestingly, there are three states, each of which harbor specific cell-mark combination from two cell types, implying similar cell types can share specific regulatory elements.

The 20 specific states have, on average, ~ 35,501 CSREs (ranging from 9554 in HepG2_3 to 77,601 in NHEK_HMEC_1; and Additional file 1: Figure S6A), spanning an average ~ 1% of the genome. The median lengths of CSREs across the 20 states were similar (around 600 bp), except two of them (1200 bp for H1_3 and 2200 for HepG2_3) are longer than the others (Additional file 1: Figure S6B). The genome covered by specific states, varies from ~ 10.5 (HSMM_NHLF) to 51.9 Mb (NHEK_HMEC_1) (Additional file 1: Figure S6C). The number of CSRE proximal genes also varies, from 284 (HSMM_1) to 3459 (HepG2_3) (Additional file 1: Figure S6D). The diversity of those statistics may indicate the functional complexity of those specific states.

### Specific states relate to various genomic features

We next explored the relationship between CSREs from different specific states and six genomic features. The proportion and fold change of CSREs in genomic features varies across different specific states (Fig. 2b, and Additional file 1: Figure S7 and S8). Specific states marked by H3K4me1 have more proportion of CSREs in intergenic regions and less in promoters than states with H3K4me3 in corresponding cell types, e.g. K562_1 vs K562_2, which is consistent with that H3K4me1 mainly locates in enhancers but H3K4me3 mainly centered around TSSs. H1_3, the unique state marked by H3K27me3, which is related to Polycomb-repressed region, has the highest proportion of CSREs in promoters, implying their proximal genes are tuned in poised status. Observation of this state is consistent with the characteristic of embryonic stem cells [25]. CSREs of HepG2_3 are substantially enriched in 5’UTR, 3’UTR, exon and intron when compared to those of the other specific states, which is expected as HepG2_3 has specific high H3K36me3 signals.

Even though specific states are not enriched in intergenic regions (Additional file 1: Figure S7), this group still constitutes ~ 43.6% of total CSREs on average, indicating the potential regulatory roles of non-coding regions. For the intergenic CSREs of each specific state, we calculated the distances to their nearest TSSs and found that they are significantly shorter than those of randomly simulated CSREs (Fig. 2c), suggesting they have the tendency to their nearest genes even though they do not overlap them. This implys that intergenic CSREs may regulate its nearby genes.

### CSREs demonstrate distinct functional specificity

**Fig. 3:**
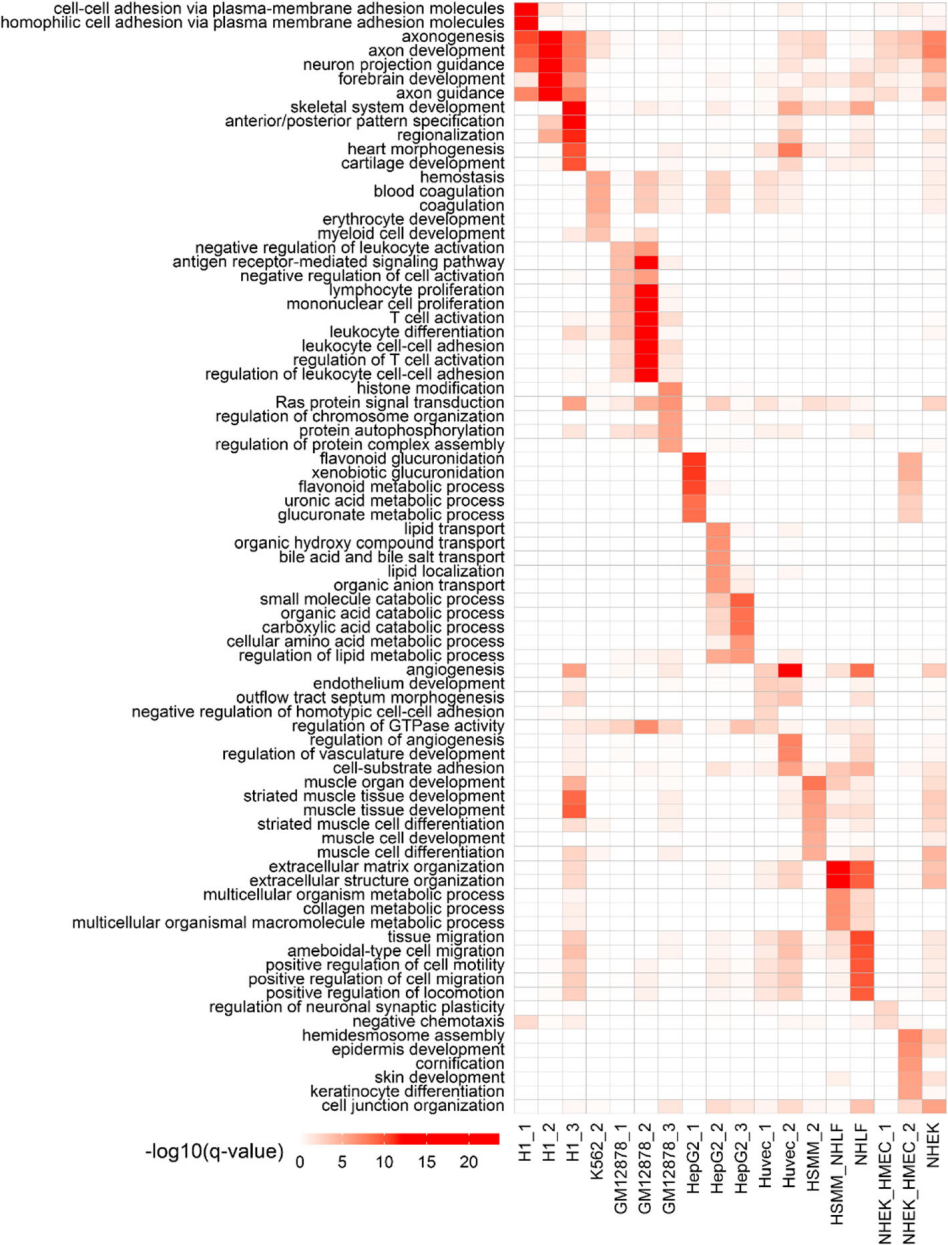
Functional enrichment of CSRE proximal genes for each specific state. Top 5 enriched GO terms with *q*-value < 0.01 for each specific state are selected, and –log_10_(*q*-value) is used to generate the heatmap

As CSREs from specific states are covered by cell type-specific histone marks, their proximal genes are expected to participate in cell type-specific functions. To verify our expectation, we conducted GO enrichment analysis for CSRE proximal genes from each specific state. We found the overrepresented GO terms were indeed highly relevant to the specific functions of corresponding cell types (Fig. 3). For example, CSRE proximal genes of HUVEC related states are enriched in terms “angiogenesis”, those of GM12878 related states are enriched in terms “lymphocyte proliferation” and “leukocyte differentiation”. Interestingly, we found that CSRE proximal genes from different specific states of a cell type can work corporately in some biological functions and can also work independently in some others. For example, CSRE proximal genes from H1_1/2/3 are all enriched in “axon development”, indicating they work collaboratively to conduct this biological function. In contrast, only genes from H1_3 are enriched in “skeletal system development” and “cartilage development”. Similarly, genes from HepG2_1 and HepG2_2 also conduct distinct functions about metabolic process and compound transport, respectively. For specific states shared by two cell types, their CSRE proximal genes are also enriched in GO terms they shared. For example, proximal genes of HSMM_NHLF are enriched in “extracellular structure organization”. Moreover, some GO terms relate to genes proximal to CSREs from diverse cell types. For example, both proximal genes of H1_3 (potential Polycomb-repressed poised regulators) and HSMM_2 (potential active regulators) are enriched in “muscle tissue development”, suggesting this function is repressed in H1 and activated in HSMM. Those results highlight the potential important roles of CSREs in regulating expression patterns of genes with cell type-specific functions.

**Fig. 4:**
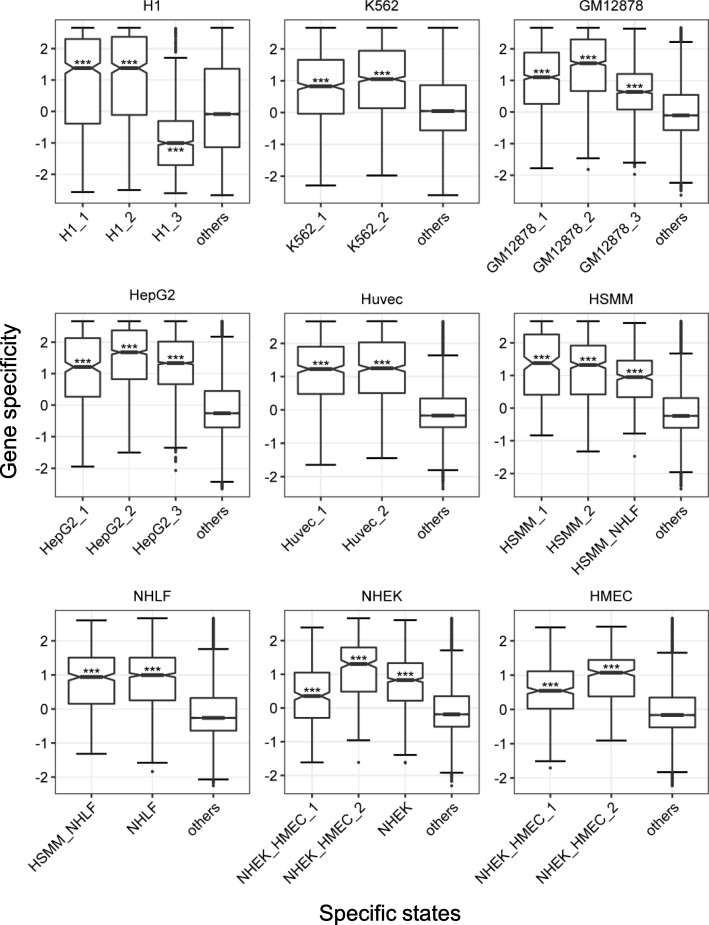
Specificity distribution of CSRE proximal genes for each specific state in the corresponding cell type. ‘others’ stands for genes not belonging to CSRE proximal genes for any specific state in the cell type. The difference between each group of CSRE proximal genes and the ‘others’ is measured by two-sample Wilcoxon test with ‘***’ indicating *P* < 0.001

If CSREs really participate in regulating its proximal genes, the expression of those genes should also be specific. To examine this assumption, we calculate the distribution of gene specificity for each group of CSRE proximal genes and the others in corresponding cell types (Fig. 4, Methods). We found that CSRE proximal genes from all specific states, except those of H1_3, have specific high expression compared with the ‘others’. Consistently, all those specific states, except H1_3, harbor active specific histone modifications (Fig. 2a). In contrast, H1_3 owns specific high Polycomb-repression mark H3K27me3 and CSRE proximal genes from this group are indeed specific low expressed as expected. Thus, for H1, there are two opposite directions of gene regulation for CSREs from different specific states. We also noticed that for 8 cell types, CSRE proximal genes from specific states with more active histone marks have higher median gene specificities, suggesting there are incremental effect of histone marks in activating cell type-specific gene expression. For specific states that are shared by two cell types, such as HSMM_NHLF, their CSRE proximal genes are specific high expressed in both cell types, implying those genes are likely to play a role in biological functions shared by both cells. We should note that genes from H1_1/2 and NHEK_HMEC_1 have low median expression compared with the ‘others’ in H1 and NHEK, respectively (Additional file 1: Figure S9). Thus, the specific high expressed genes are not necessarily the top expressed ones in a cell type, which would be easily ignored without the comparative analysis.

### Relationship between CSREs and DNase peaks or EP300 binding sites

Peaks of DNase-seq are open chromatin around where transcription factors can easily bind to DNA. DNase peaks have been comprehensively exploited to identify regulatory elements in diverse cell types [26]. Differential DNase-seq footprinting identifies cell type determining transcription factors [27]. Thus, we expected CSREs were likely to be proximal to cell type-specific DNase peaks. Indeed, CSREs from all specific states overlap significantly more peaks than the random simulated ones do (Additional file 1: Figure S10), which suggests that CSREs, as well as their underlying modifications, could play a crucial role in cell type-specific regulatory activities.

**Fig. 5:**
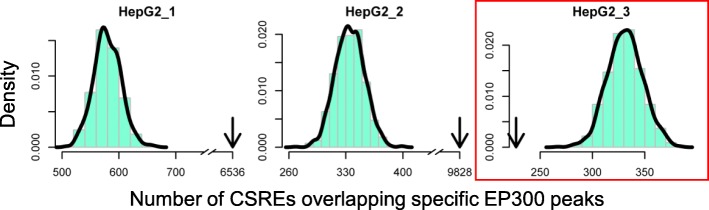
Relationship between CSREs from each specific state and cell type-specific EP300 binding sites in HepG2. The histograms show the distributions of number of random simulated CSREs overlapping cell type-specific EP300 peaks, and the curves are plotted based on kernel density estimate. The arrows indicate the number of overlaps for true CSREs. Statistical significance is calculated by one-sample Wilcoxon test with *P* < 2.2e–16 for all cases

EP300 is a transcriptional co-activator and lineage-specific EP300 peaks has been used to identify cell type-specific transcriptional enhancers [28]. We found that CSREs from all specific states, except HepG2_3, overlap significantly more EP300 peaks than the random simulated ones do in corresponding cell types (Fig. 5 and Additional file 1: Figure S11), indicating many CSREs in those states are adjacent to enhancers. Interestingly, even CSREs from H1_3 are covered by repressive mark H3K27me3, they are still enriched in EP300 peaks, suggesting many of them are poised enhancers [29]. In contrast, HepG2_3 is marked by the specific H3K36me3 and its CSREs locate largely in gene bodies (Fig. 2a and b). Thus, those CSREs are expected to be on bodies of highly expressed genes, and not likely to be enhancers, which is consistent with our observation.

### CSREs reveal cell type-specific behavior of genes: Two case studies

**Fig. 6:**
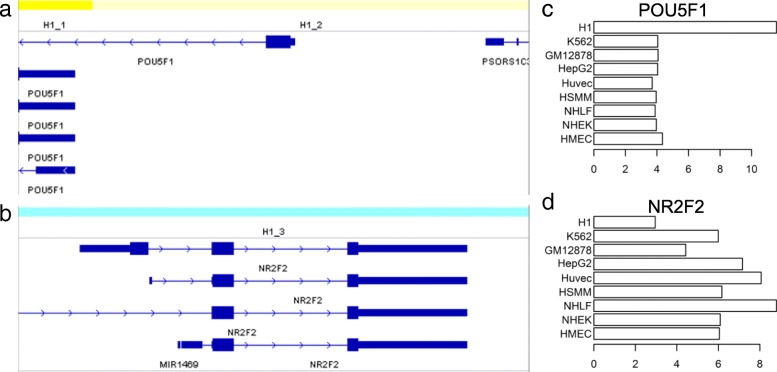
Illustration of CSREs covering POU5F1 and NR2F2, respectively. **a** CSREs from H1_1 and H1_2 overlapping POU5F1. **b** CSRE from H1_3 overlapping NR2F2. **c** Expression of POU5F1. **d** Expression of NR2F2

As aforementioned, CSREs may regulate genes in different directions in a cell type, which was not shown by dCMA. To explicitly illustrate that, we took H1 embryonic stem cells as an example. POU5F1, also known as OCT4, is a well-known marker gene of human embryonic stem cells (hESCs). It is essential for maintaining the self-renewing undifferentiated state of hESCs [30]. A previous study showed that POU5F1 repress NR2F2 at the transcriptional level in the undifferentiated state [31]. Interestingly, in the H1 hESC cell line, both POU5F1 and NR2F2 contain CSREs, but from different specific states (Fig. 6a and b). Specifically, promoters of POU5F1 isoforms are covered by CSREs belonging to either H1_1 or H1_2. The CSRE of H1_1 contains peaks of H3K4me1/2 unique to this cell line, and that of H1_2 harbors additional specific histone modifications, including H3K27ac, H3K9ac and H3K4me3 (Additional file 1: Figure S12 and S13), which is consistent with the emission probability profile of the two states (Fig. 2). As both CSREs are covered by combinations of active histone modifications, their proximal genes may be upregulated. Consistently, POU5F1 has a pronounced expression against the other cell types examined (Fig. 6c). In contrast, three NR2F2 isoforms are encompassed by a CSRE of H1_3, which contains specific H3K27me3 peaks and lacks of H3K27ac and H3K9ac compared with other cell types (Additional file 1: Figure S14 and S15). As aforementioned, H3K27me3 marks Polycomb-repressed region [32]. Thus, we expects NR2F2 to be in a poised status, and the expression of NR2F2 is indeed relatively lower than the other cell types (Fig. 6d). These distinctive chromatin modification patterns highlight specialized epigenomic regulation of these two genes, which can be precisely revealed by the subclasses of CSREs in this cell type.

For the CSREs shared by two cell types, we expected that their proximal genes also function specifically in both cell types. We took a CSRE in NHEK_HMEC_2 as an example. We found that the third longest CSRE of NHEK_HMEC_2 is a 9600-bp region encompassing the whole gene body of KRT14 (Additional file 1: Figure S16). This gene provides instructions for making keratin 14, which is a fibrous protein making up skin [33]. Besides, it was also known as an epithelial marker [34]. As expected, we observed strikingly expressed KRT14 in both NHEK and HMEC (Additional file 1: Figure S17B). Intriguingly, in the two cell types, more than 3/4 of the CSRE harbors active marks H3K27ac, H3K9ac and H3K4me1/2, which are nearly empty in this region among the other cell types (Additional file 1: Figure S17A), indicating KRT14 may be regulated by the precise histone modification pattern in both cell types.

### Application of CsreHMM to a large-scale dataset reveals hierarchical specific CSREs

We also applied CsreHMM to a large-scale dataset of 127 cell types and tissues from the Roadmap Epigenomics Project [4] (Additional file 2: Table S1). Some of these cell types or tissues come from the same lineage and are very similar to each other. As the difference of cell types from different lineages would be larger than that of cell types within the same lineage, directly applying CsreHMM to this dataset would more likely focus on difference between lineages and lead to lineage-specific chromatin modified region.

**Fig. 7:**
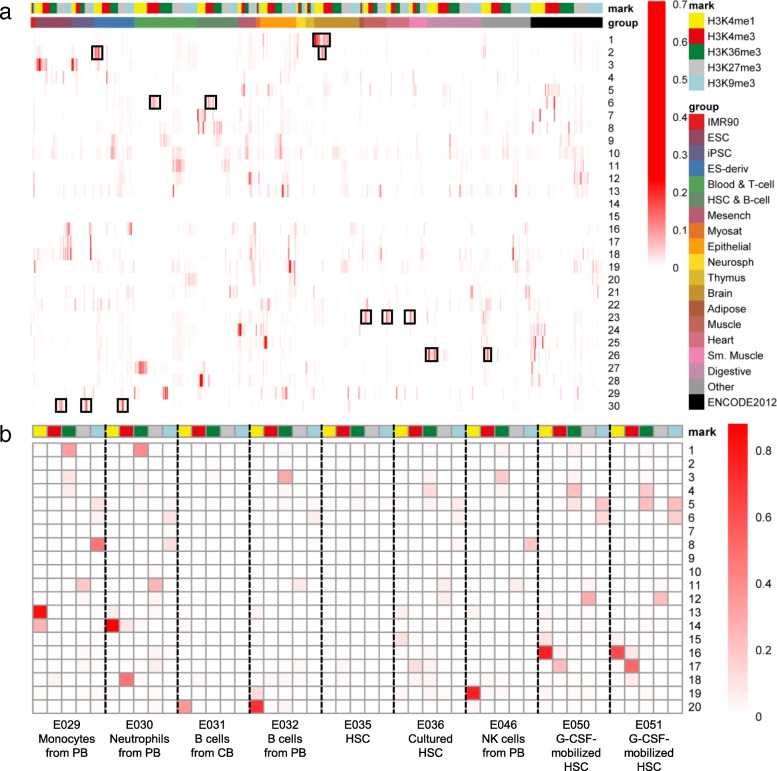
Emission probabilities of (**a**) a 30-state model for Roadmap Epigenomics dataset and (**b**) a 20-state model for group “HSC & B cell”. Each column stands for a cell-mark combination. The group information comes from Roadmap Epigenomics Consortium. Exxx stands for the EID for each cell type or tissue (Additional file 2: Table S1). PB, peripheral blood; CB, cord blood; HSC, hematopoietic stem cells; NK cells, nature killer cells

In practice, we trained a 30-state model for the whole dataset. As expected, we found that some states are specific regions shared by cell types from the same lineage, which could be traited as lineage-specific regulatory elements. For example, state 1 obtains specific H3K4me1 and H3K4me3 signal of multiple brain tissues (Fig. 7a), and their proximal genes are significantly enriched in GO term “learning” and “cognition” (Additional file 2: Table S2), which implies this state consists of regulatory elements specific for brain. In additation, we also found that state 6, 23, 26, 30 relate to blood-, muscle-, liver- and ES-specific regulatory elements, respectively (Fig. 7a and Additional file 2: Table S2). Interestingly, we noticed that state 2 are associated with both fetal brain and ES-derived neuron cells, and their proximal genes are significantly enriched in GO term “axon development” and “axonogenesis”, which indicates that this state covers region that may play an important role in brain development.

Even though it is hard to focus on the difference within a lineage by directly applying CsreHMM to the whole dataset, we can still achieve it by applying the model to epigenomics within a specific lineage. For example, we trained a 20-state model for 9 cell types in group “HSC & B cell”, to see the subtle difference among them (Fig. 7b). We found that state 1, 14 and 18 has neutrophils-specific H3K36me3, H3K4me1 and H3K4me3 signal, respectively, indicating that they are activate regulators of neutrophils. Surprisingly, for all the 3 states, their proximal genes are significantly enriched in GO term “neutrophil activation” (Additional file 2: Table S3). State 19 obtains nature killer cell-specific H3K4me1 signal. Interestingly, its proximal genes are significantly enriched in “T cell activation” (Additional file 2: Table S3), which seems surprising but is consistent with recent observation that NK cells contribute to the activation of T cells [35].

In summary, this application demonstrates the ability of CsreHMM to find lineage- or cell type-specific regulatory elements from large-scale epigenomic data.

## Discussion

Here we introduced a comparative computational method CsreHMM to systematically identify cell type-specific regulatory elements along the whole genome and their characterized histone codes. We applied our method to a ENCODE dataset and found that two thirds of states from the trained HMM model were identified as specific ones, illustrating its efficiency in revealing more detailed regulatory characteristic. The identified CSREs were enriched in different kinds of regulatory regions; their proximal genes were likely to participate in cell type-specific biological functions; the expressions of those genes were also in line with the underlying histone codes of their proximal CSREs. All those results demonstrate the effectiveness of our method.

Compared with dCMA, CsreHMM can not only locate CSREs for each cell type, but also identify the mark combinations that characterize their specificity and reveal their sub-patterns and explicitly label the CSREs shared by multiple cells. Those additional features can bring us a more deep understanding of CSREs. However, the limited number of states can only capture recurrent types of CSREs, consequently omitting the rare ones, which can be picked up by carefully examining the histone codes of each CSRE provided by dCMA. Thus, CsreHMM is more suitable to get a general picture of CSREs to help understand specific behaviors of histone modifications in a cell type and the formation of cell identity.

Large epigenomic datasets usually contain cell types from the same lineage. When applied to such a dataset, CsreHMM would be more likely to find lineage-specific regulatory elements, rather than cell type-specific ones. Even thouth increasing the number of states would grasp subtle difference between cell types within a lineage, and may discover the cell type-specific regulatory elements, this procedure would also increase the training time quadratically. Instead, we suggest to apply CsreHMM to a specific lineage of cell types to make the comparison more reasonable and make the cost much lower.

With the continuous generation of more genome-wide epigenomic data by large consortium like IHEC [36], we expect this method to become a useful tool for investigating diverse chromatin modifications among multiple cell types or conditions.

## Additional files

Additional file 1: Supplementary Figures. (PPTX 6008 kb)

Additional file 2: Supplementary Tables. (XLSX 44 kb)

Additional file 3: Bed file of all CSREs. (ZIP 9707 kb)
