# Supplementary material for: Reveal cell type-specific regulatory elements and their characterized histone code classes via a hidden Markov model
Source: BMC Genomics. 2018 Dec 31;19(Suppl 10):903. doi: 10.1186/s12864-018-5274-9 (PMC6311906; doi:10.1186/s12864-018-5274-9)
Supplement: Supplementary file 1 — Supplementary Figures. (PPTX 6008 kb) [file 12864_2018_5274_MOESM1_ESM.pptx]

## Slide 1
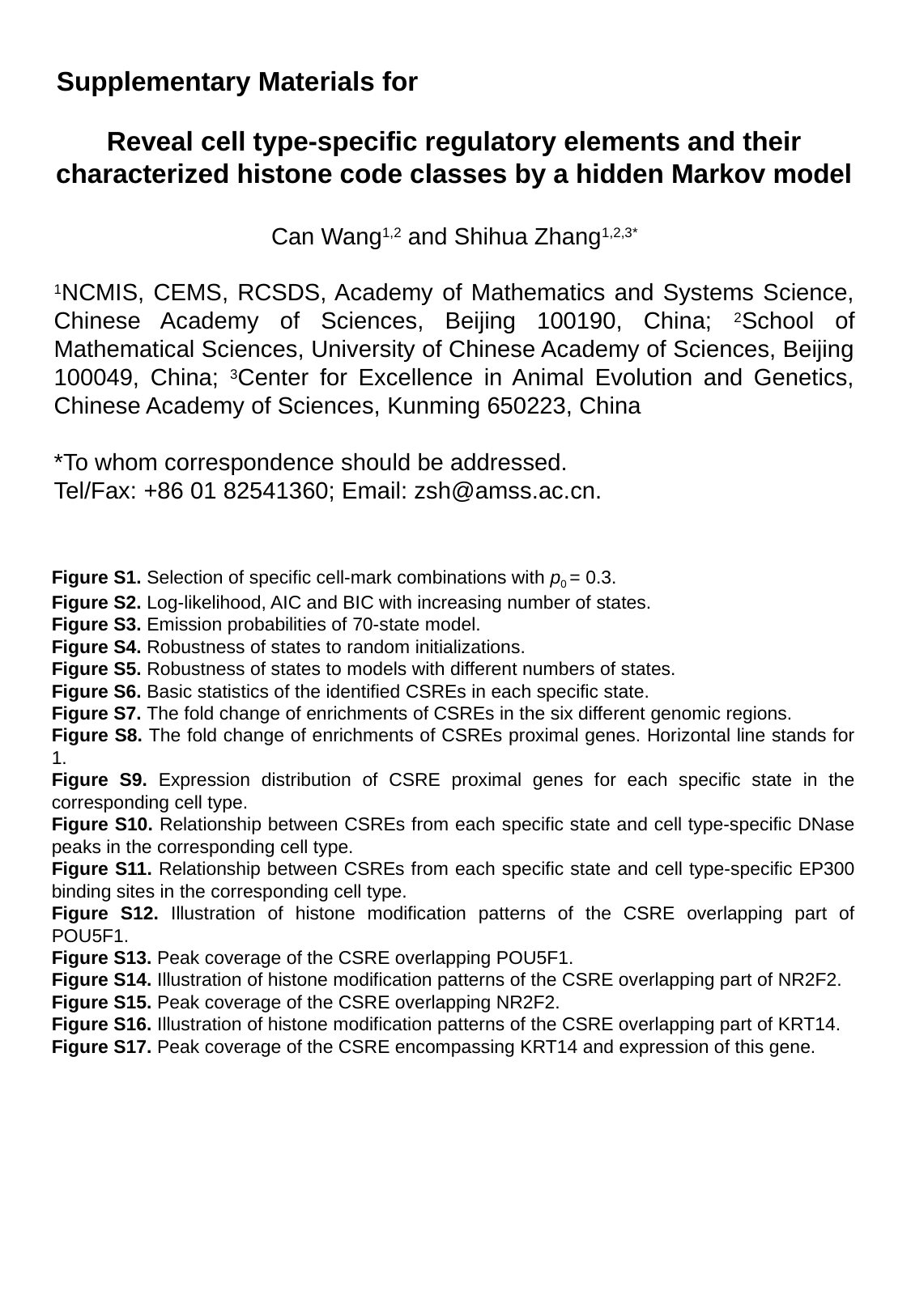

Supplementary Materials for
Reveal cell type-specific regulatory elements and their characterized histone code classes by a hidden Markov model
Can Wang1,2 and Shihua Zhang1,2,3*
1NCMIS, CEMS, RCSDS, Academy of Mathematics and Systems Science, Chinese Academy of Sciences, Beijing 100190, China; 2School of Mathematical Sciences, University of Chinese Academy of Sciences, Beijing 100049, China; 3Center for Excellence in Animal Evolution and Genetics, Chinese Academy of Sciences, Kunming 650223, China
*To whom correspondence should be addressed.
Tel/Fax: +86 01 82541360; Email: zsh@amss.ac.cn.
Figure S1. Selection of specific cell-mark combinations with p0 = 0.3.
Figure S2. Log-likelihood, AIC and BIC with increasing number of states.
Figure S3. Emission probabilities of 70-state model.
Figure S4. Robustness of states to random initializations.
Figure S5. Robustness of states to models with different numbers of states.
Figure S6. Basic statistics of the identified CSREs in each specific state.
Figure S7. The fold change of enrichments of CSREs in the six different genomic regions.
Figure S8. The fold change of enrichments of CSREs proximal genes. Horizontal line stands for 1.
Figure S9. Expression distribution of CSRE proximal genes for each specific state in the corresponding cell type.
Figure S10. Relationship between CSREs from each specific state and cell type-specific DNase peaks in the corresponding cell type.
Figure S11. Relationship between CSREs from each specific state and cell type-specific EP300 binding sites in the corresponding cell type.
Figure S12. Illustration of histone modification patterns of the CSRE overlapping part of POU5F1.
Figure S13. Peak coverage of the CSRE overlapping POU5F1.
Figure S14. Illustration of histone modification patterns of the CSRE overlapping part of NR2F2.
Figure S15. Peak coverage of the CSRE overlapping NR2F2.
Figure S16. Illustration of histone modification patterns of the CSRE overlapping part of KRT14.
Figure S17. Peak coverage of the CSRE encompassing KRT14 and expression of this gene.

## Slide 2
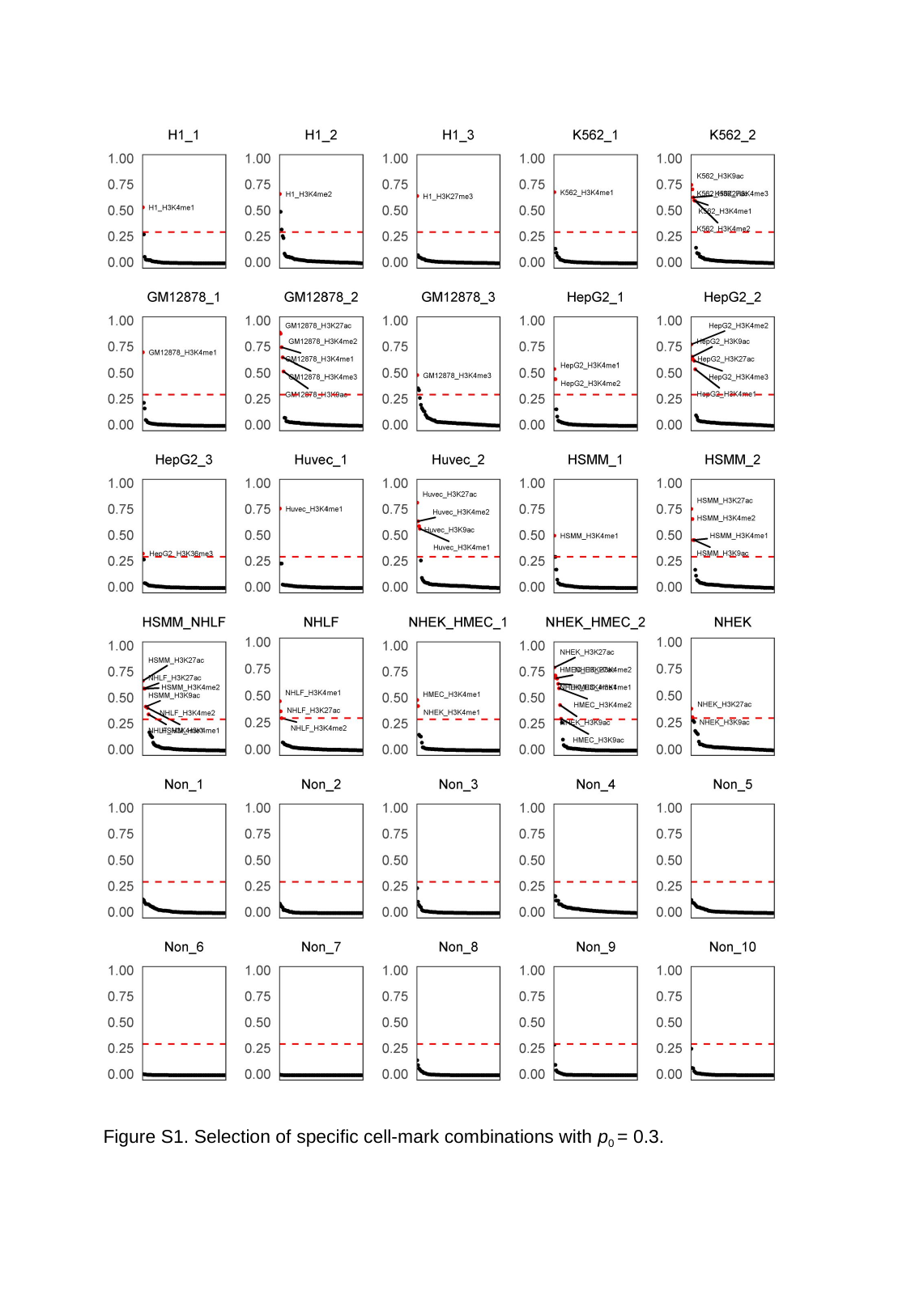

Figure S1. Selection of specific cell-mark combinations with p0 = 0.3.

## Slide 3
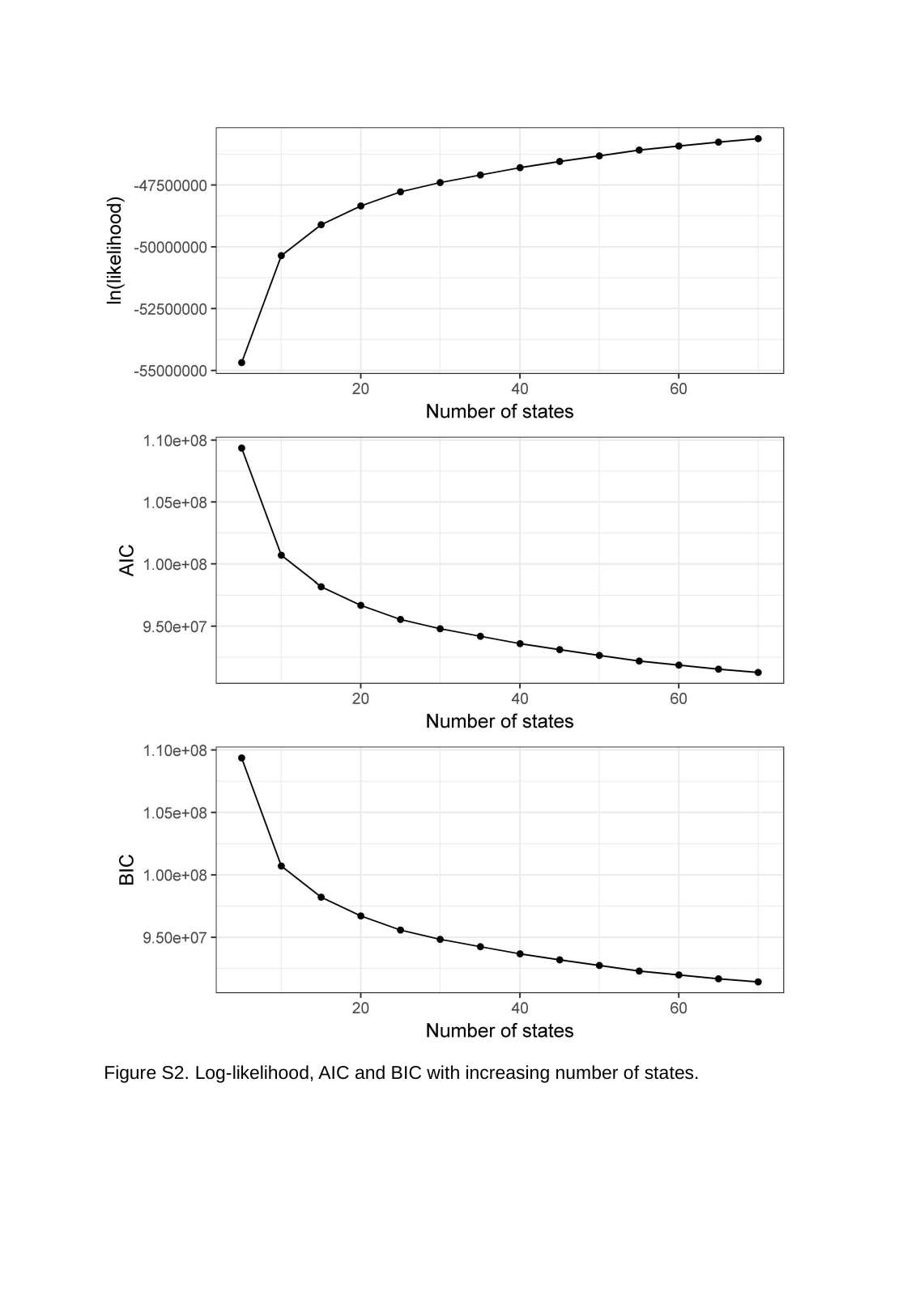

Figure S2. Log-likelihood, AIC and BIC with increasing number of states.

## Slide 4
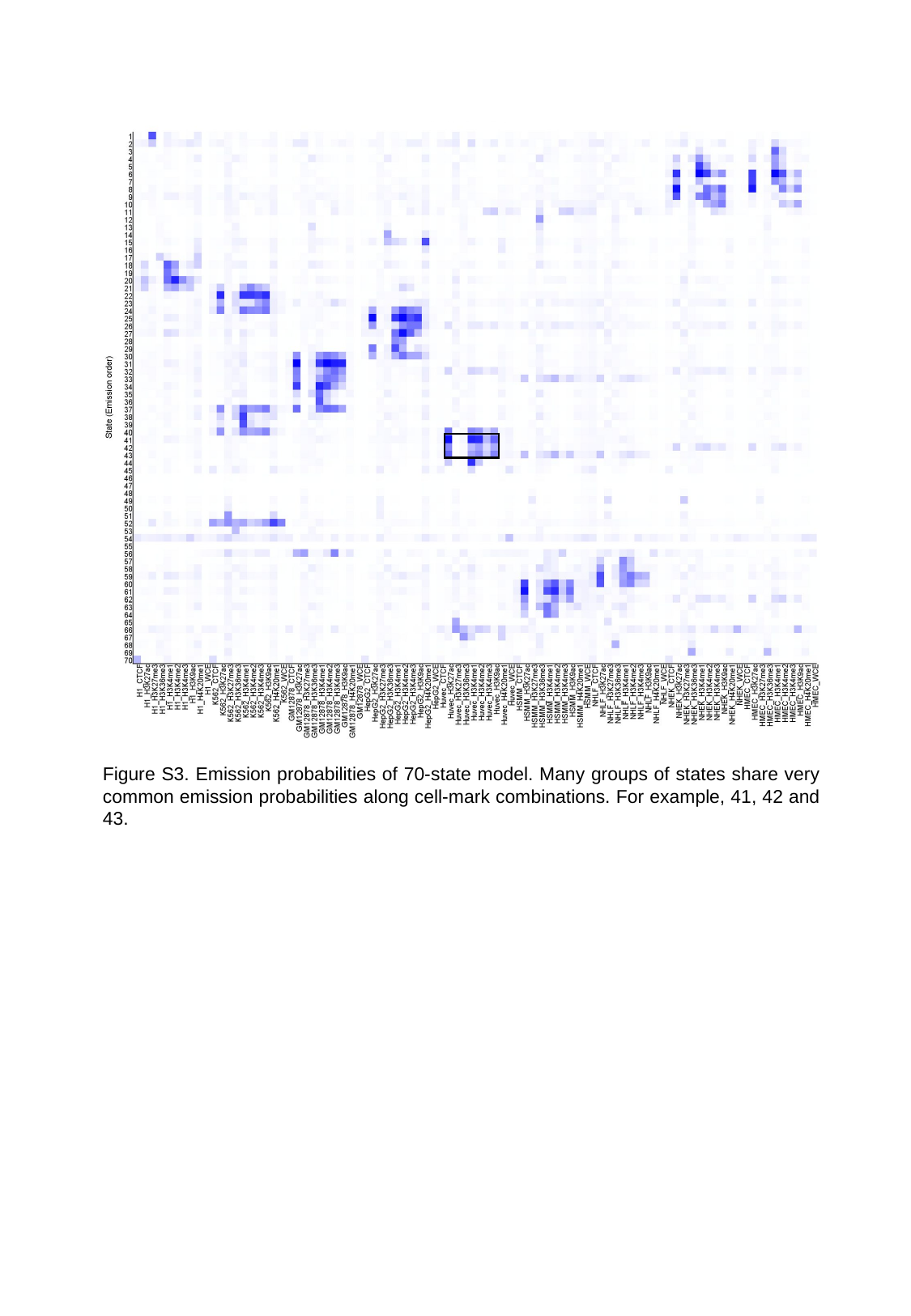

Figure S3. Emission probabilities of 70-state model. Many groups of states share very common emission probabilities along cell-mark combinations. For example, 41, 42 and 43.

## Slide 5
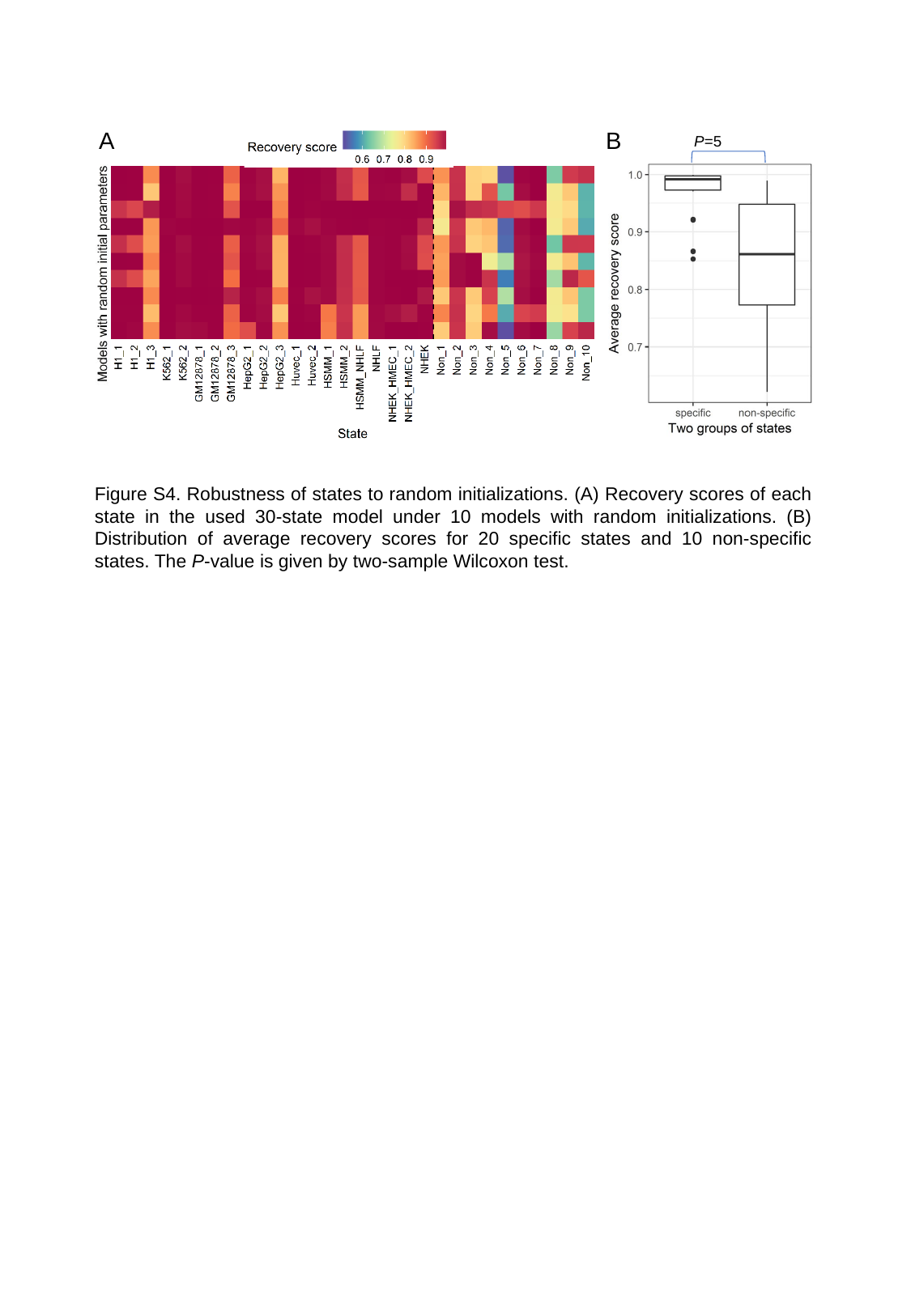

A
B
Figure S4. Robustness of states to random initializations. (A) Recovery scores of each state in the used 30-state model under 10 models with random initializations. (B) Distribution of average recovery scores for 20 specific states and 10 non-specific states. The P-value is given by two-sample Wilcoxon test.

## Slide 6
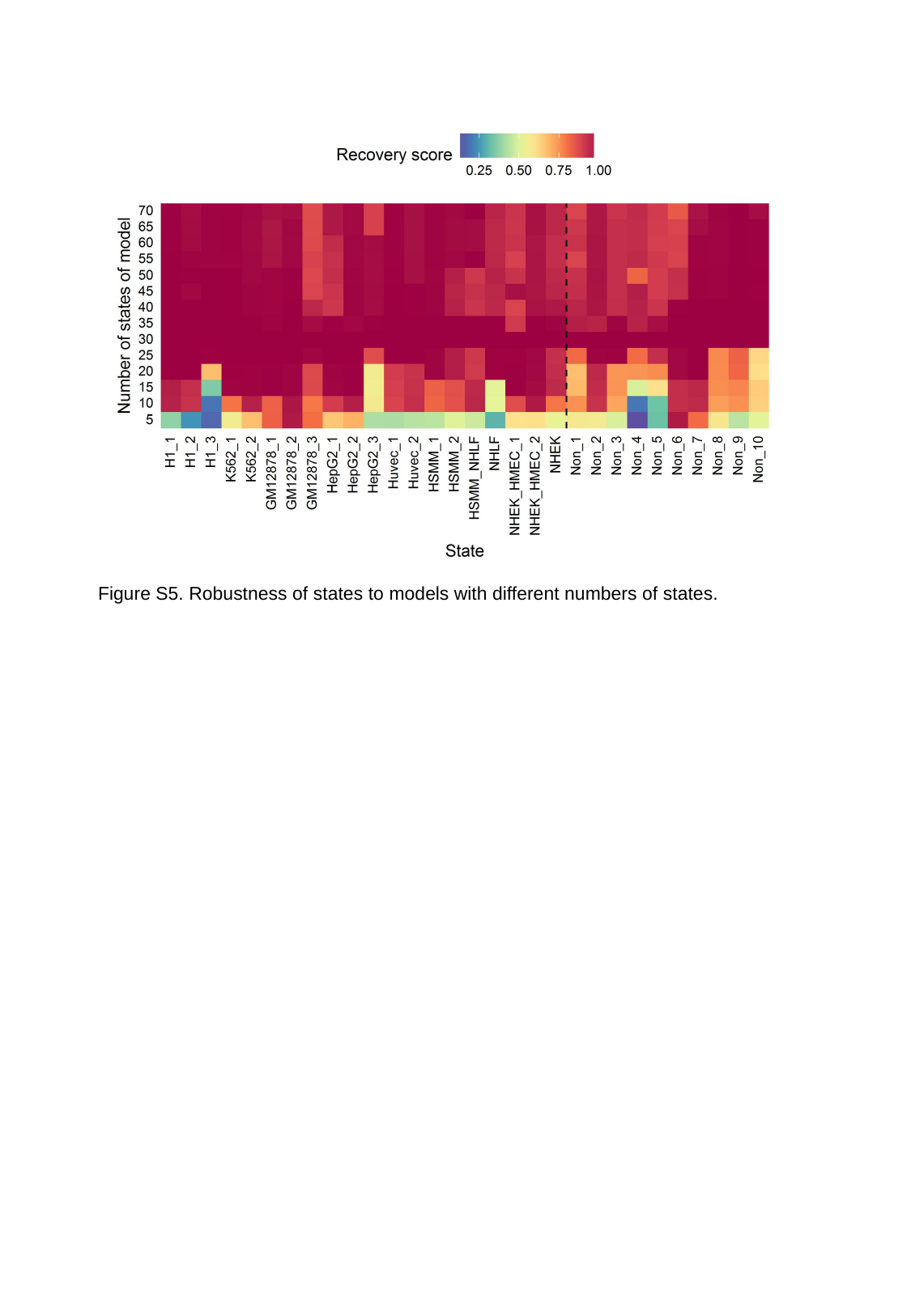

Figure S5. Robustness of states to models with different numbers of states.

## Slide 7
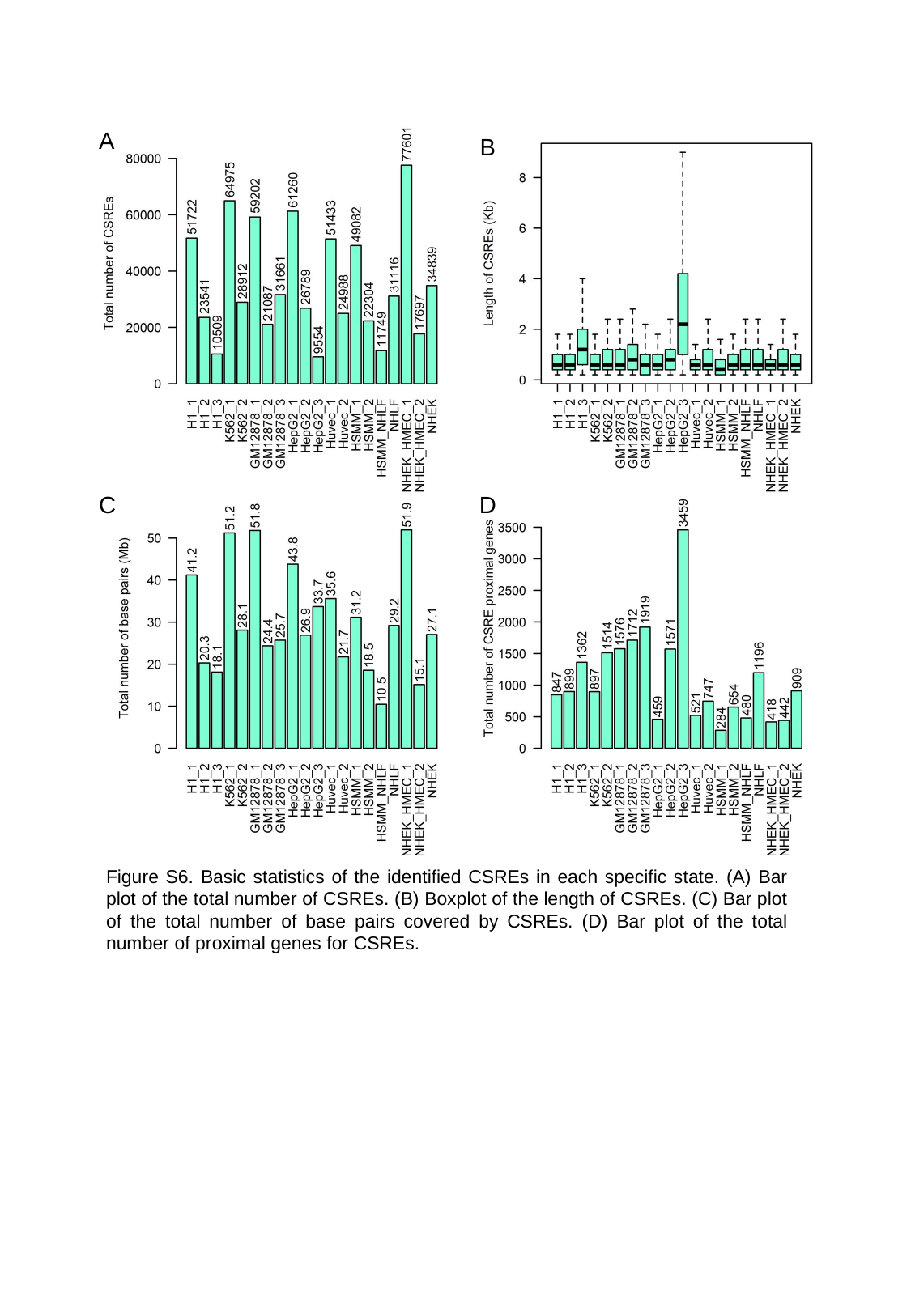

A
B
D
C
Figure S6. Basic statistics of the identified CSREs in each specific state. (A) Bar plot of the total number of CSREs. (B) Boxplot of the length of CSREs. (C) Bar plot of the total number of base pairs covered by CSREs. (D) Bar plot of the total number of proximal genes for CSREs.

## Slide 8
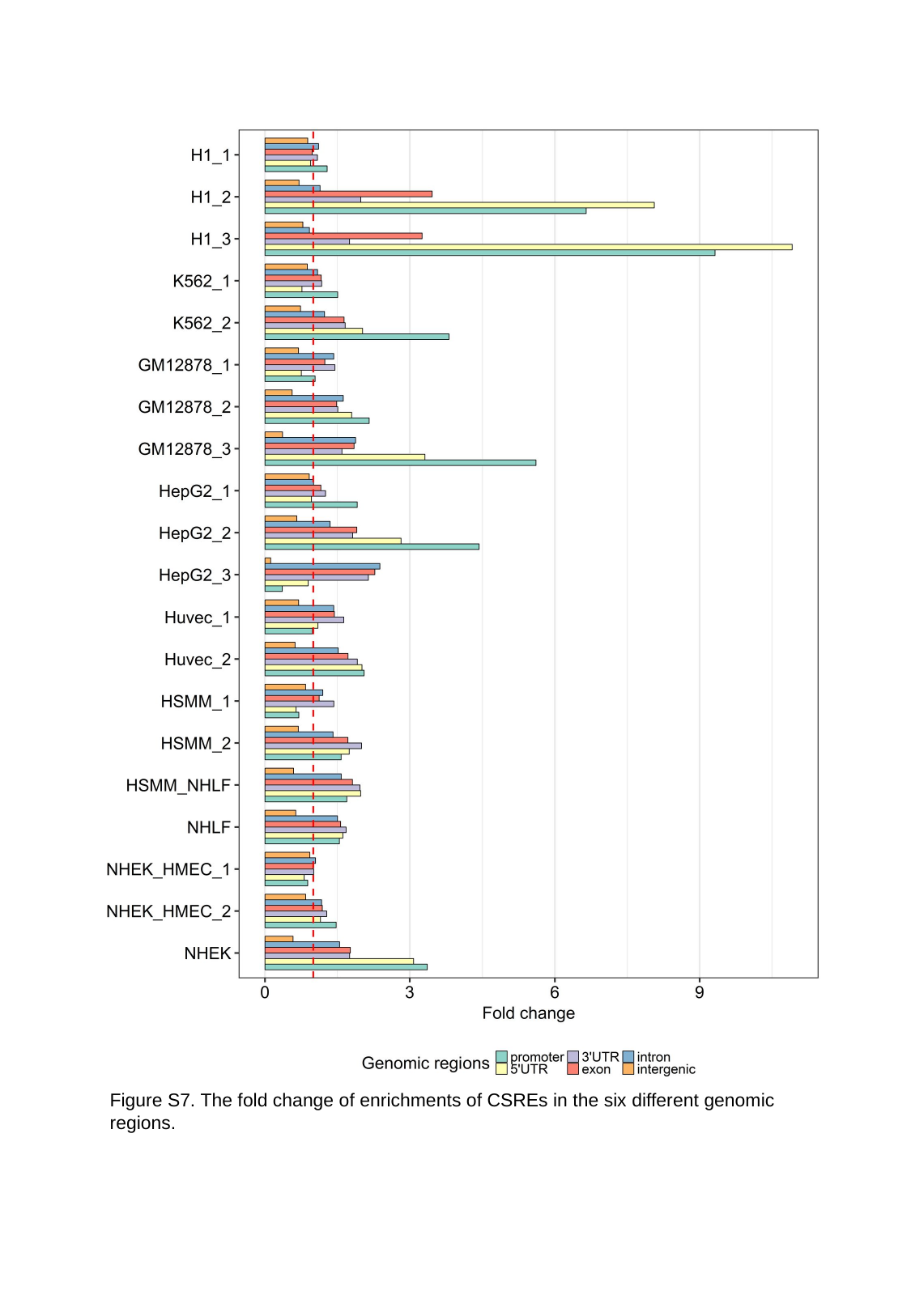

Figure S7. The fold change of enrichments of CSREs in the six different genomic regions.

## Slide 9
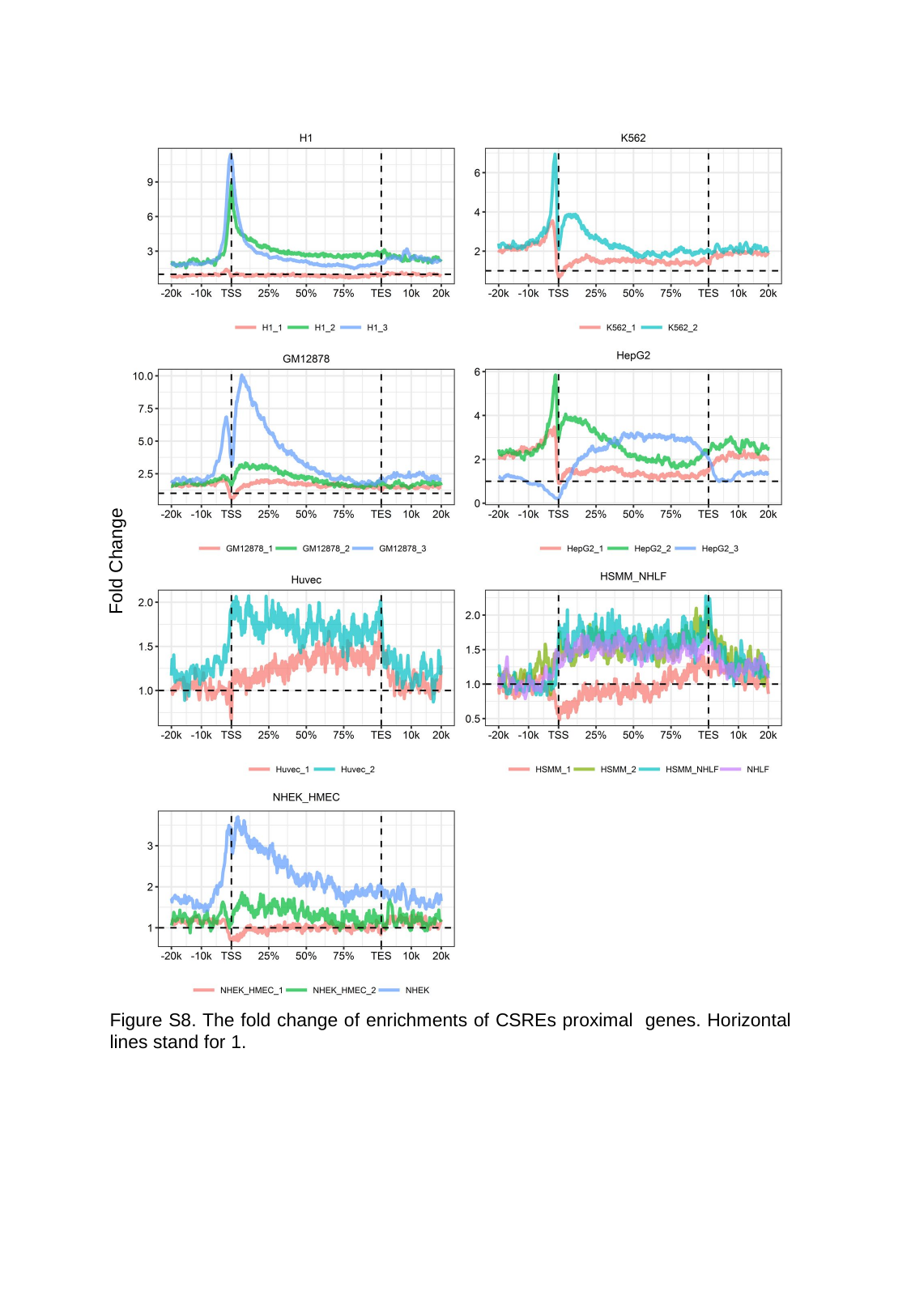

Fold Change
Figure S8. The fold change of enrichments of CSREs proximal genes. Horizontal lines stand for 1.

## Slide 10
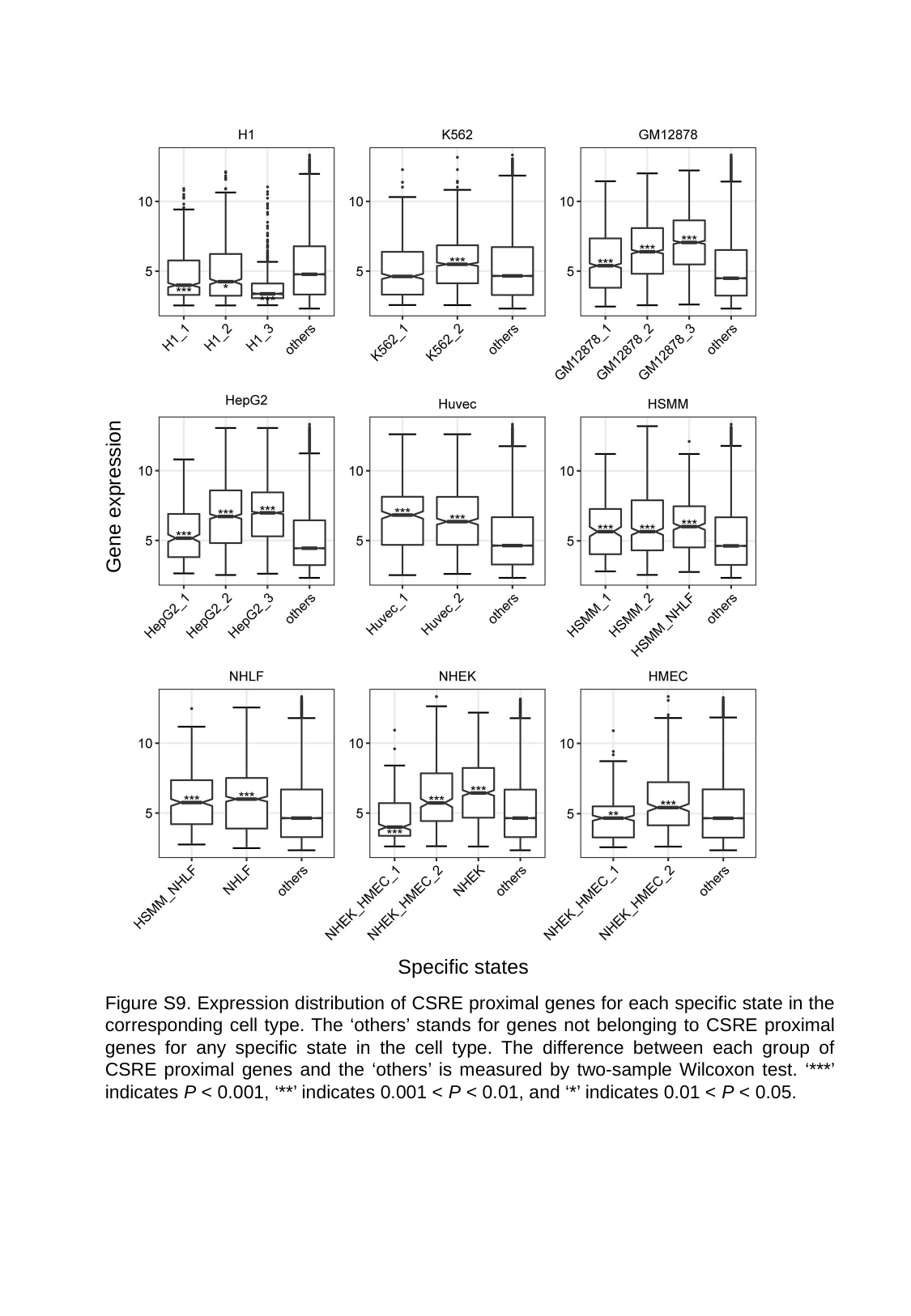

Gene expression
Specific states
Figure S9. Expression distribution of CSRE proximal genes for each specific state in the corresponding cell type. The ‘others’ stands for genes not belonging to CSRE proximal genes for any specific state in the cell type. The difference between each group of CSRE proximal genes and the ‘others’ is measured by two-sample Wilcoxon test. ‘***’ indicates P < 0.001, ‘**’ indicates 0.001 < P < 0.01, and ‘*’ indicates 0.01 < P < 0.05.

## Slide 11
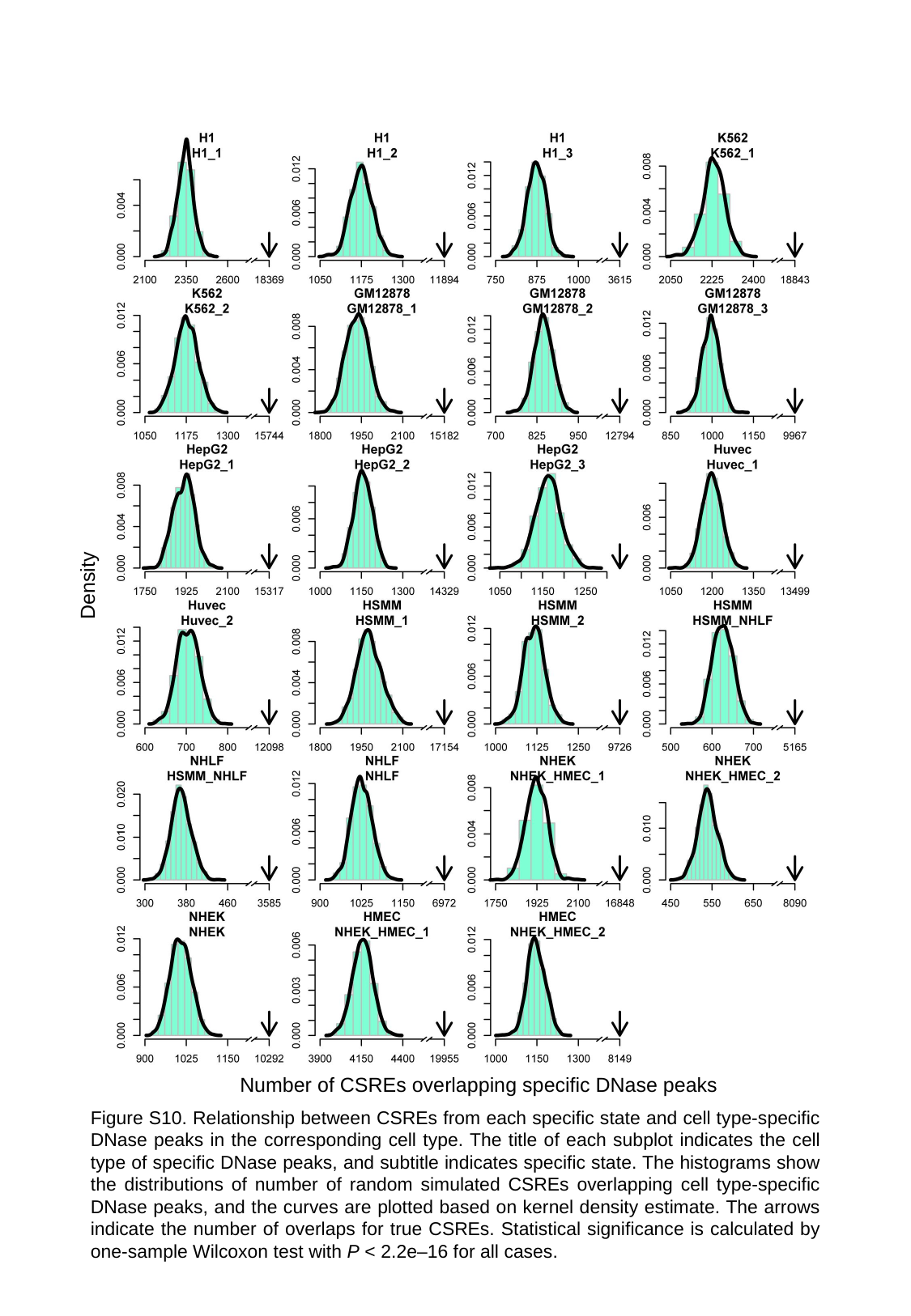

Density
Number of CSREs overlapping specific DNase peaks
Figure S10. Relationship between CSREs from each specific state and cell type-specific DNase peaks in the corresponding cell type. The title of each subplot indicates the cell type of specific DNase peaks, and subtitle indicates specific state. The histograms show the distributions of number of random simulated CSREs overlapping cell type-specific DNase peaks, and the curves are plotted based on kernel density estimate. The arrows indicate the number of overlaps for true CSREs. Statistical significance is calculated by one-sample Wilcoxon test with P < 2.2e–16 for all cases.

## Slide 12
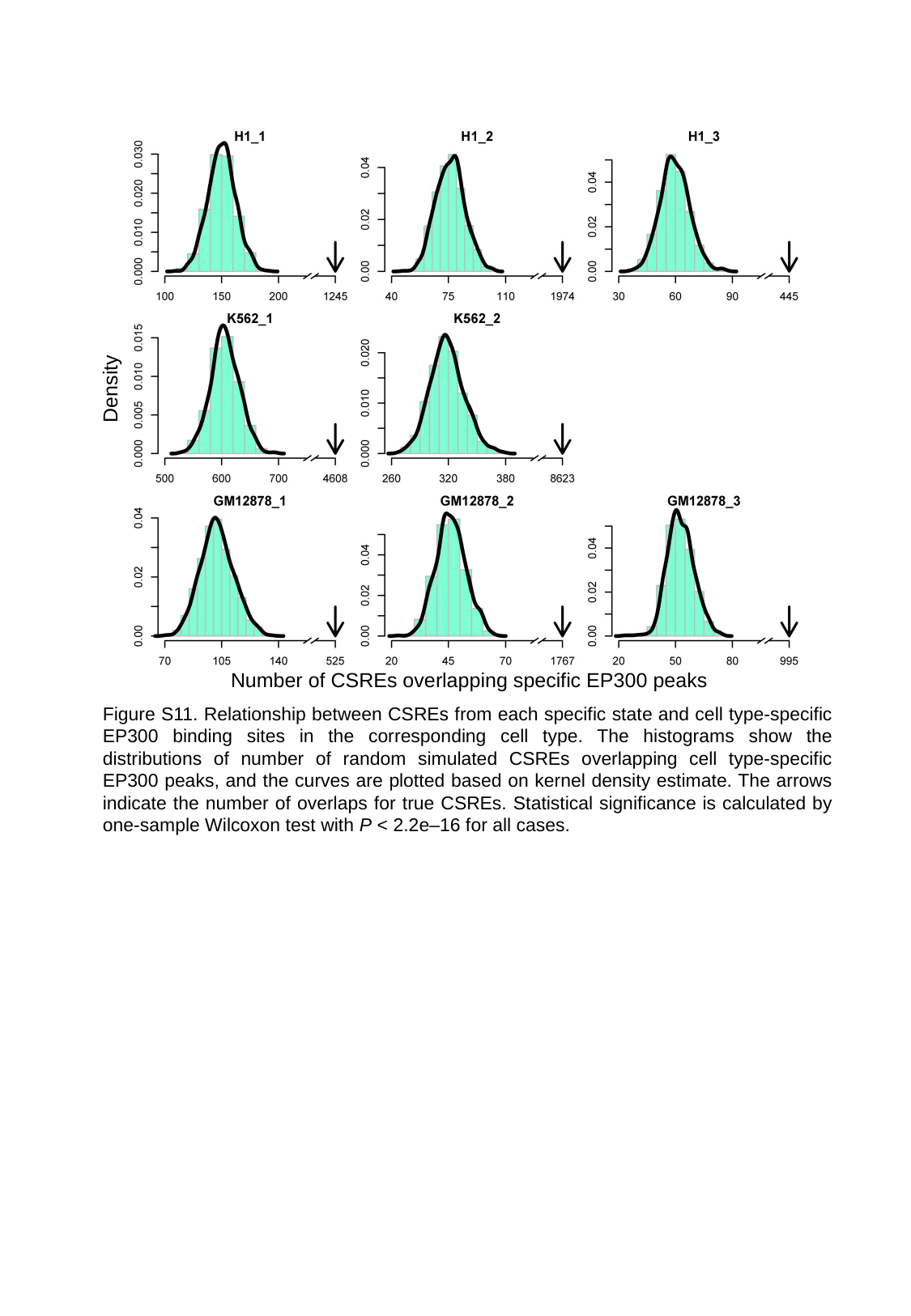

Density
Number of CSREs overlapping specific EP300 peaks
Figure S11. Relationship between CSREs from each specific state and cell type-specific EP300 binding sites in the corresponding cell type. The histograms show the distributions of number of random simulated CSREs overlapping cell type-specific EP300 peaks, and the curves are plotted based on kernel density estimate. The arrows indicate the number of overlaps for true CSREs. Statistical significance is calculated by one-sample Wilcoxon test with P < 2.2e–16 for all cases.

## Slide 13
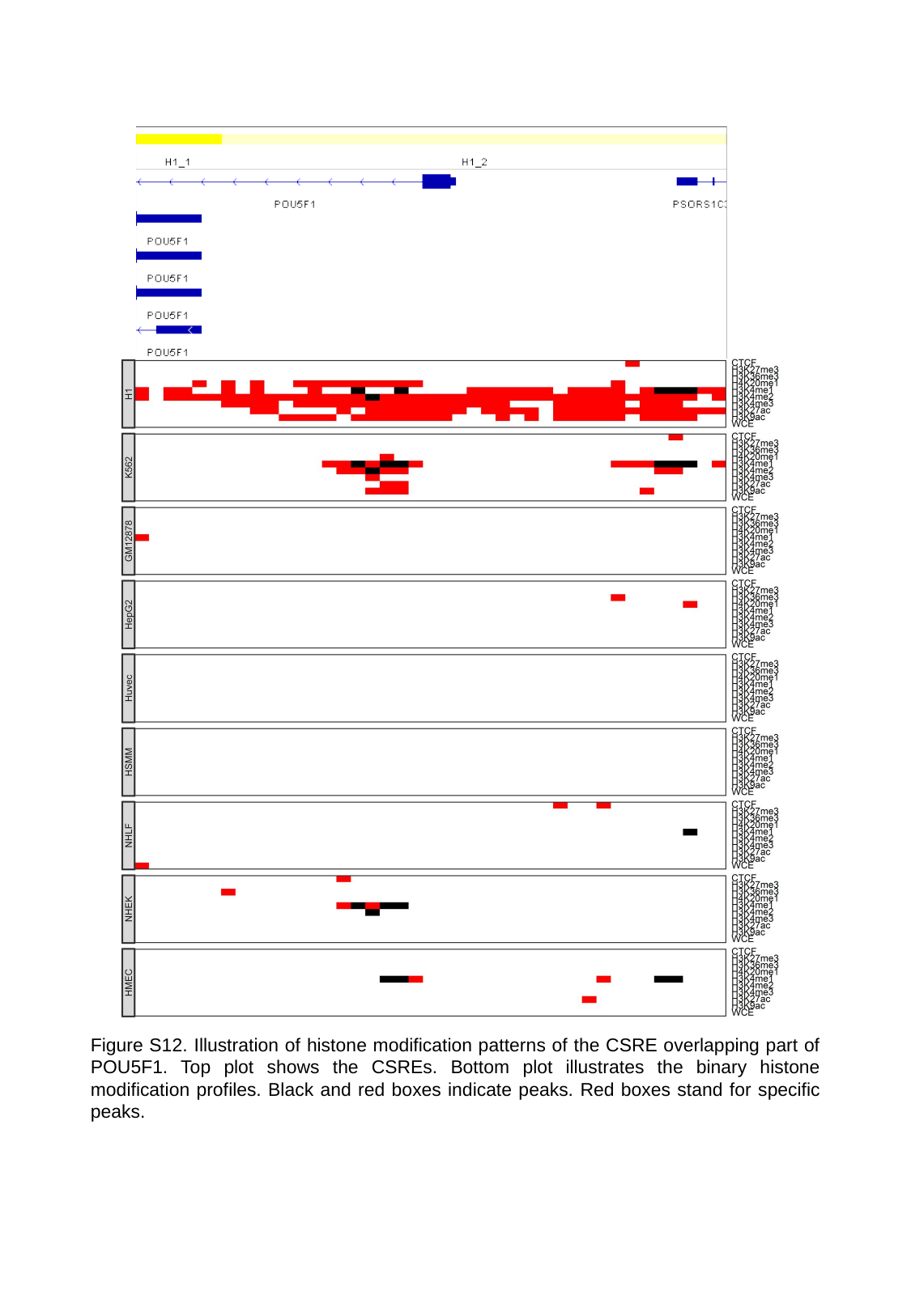

Figure S12. Illustration of histone modification patterns of the CSRE overlapping part of POU5F1. Top plot shows the CSREs. Bottom plot illustrates the binary histone modification profiles. Black and red boxes indicate peaks. Red boxes stand for specific peaks.

## Slide 14
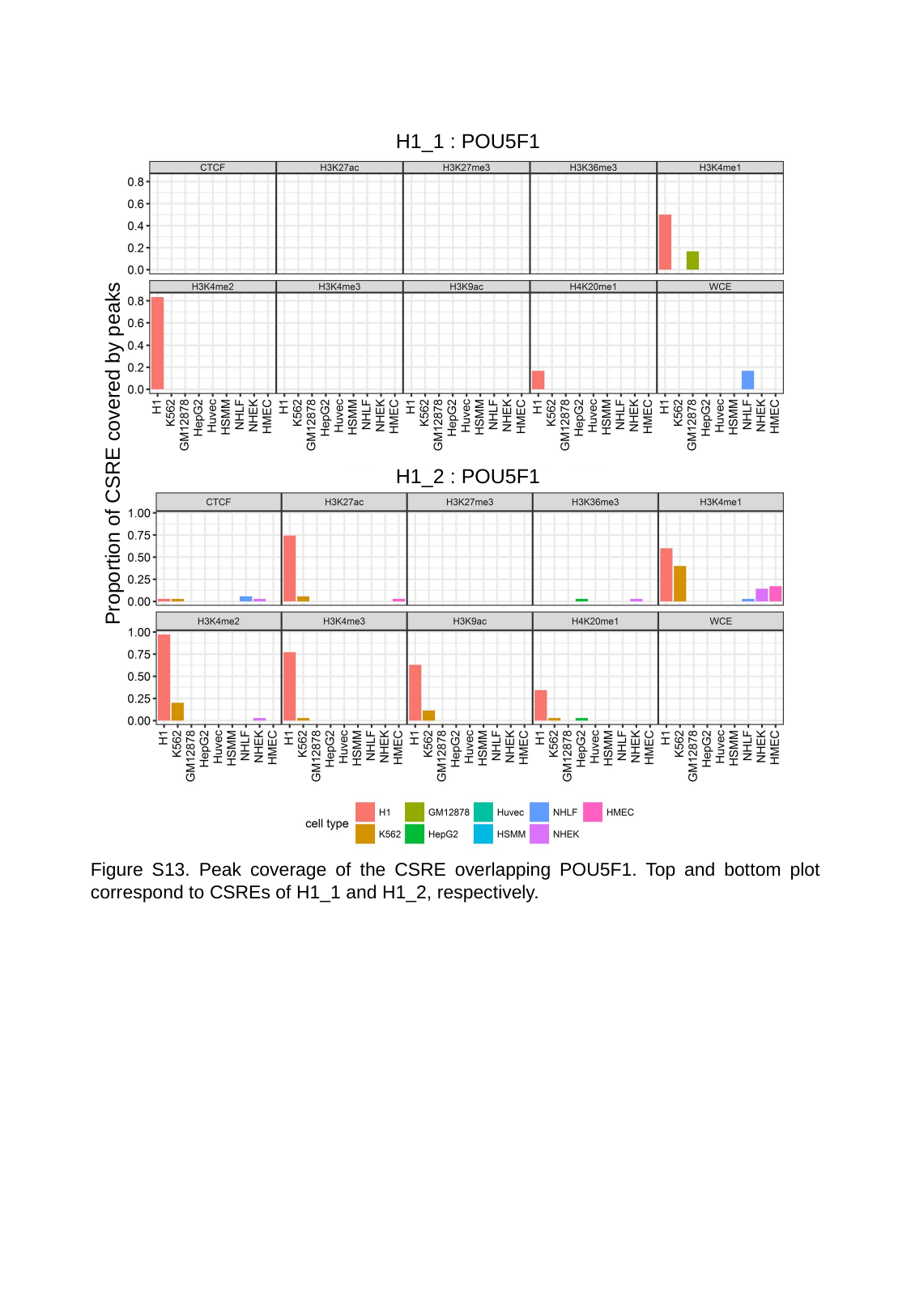

H1_1 : POU5F1
Proportion of CSRE covered by peaks
H1_2 : POU5F1
Figure S13. Peak coverage of the CSRE overlapping POU5F1. Top and bottom plot correspond to CSREs of H1_1 and H1_2, respectively.

## Slide 15
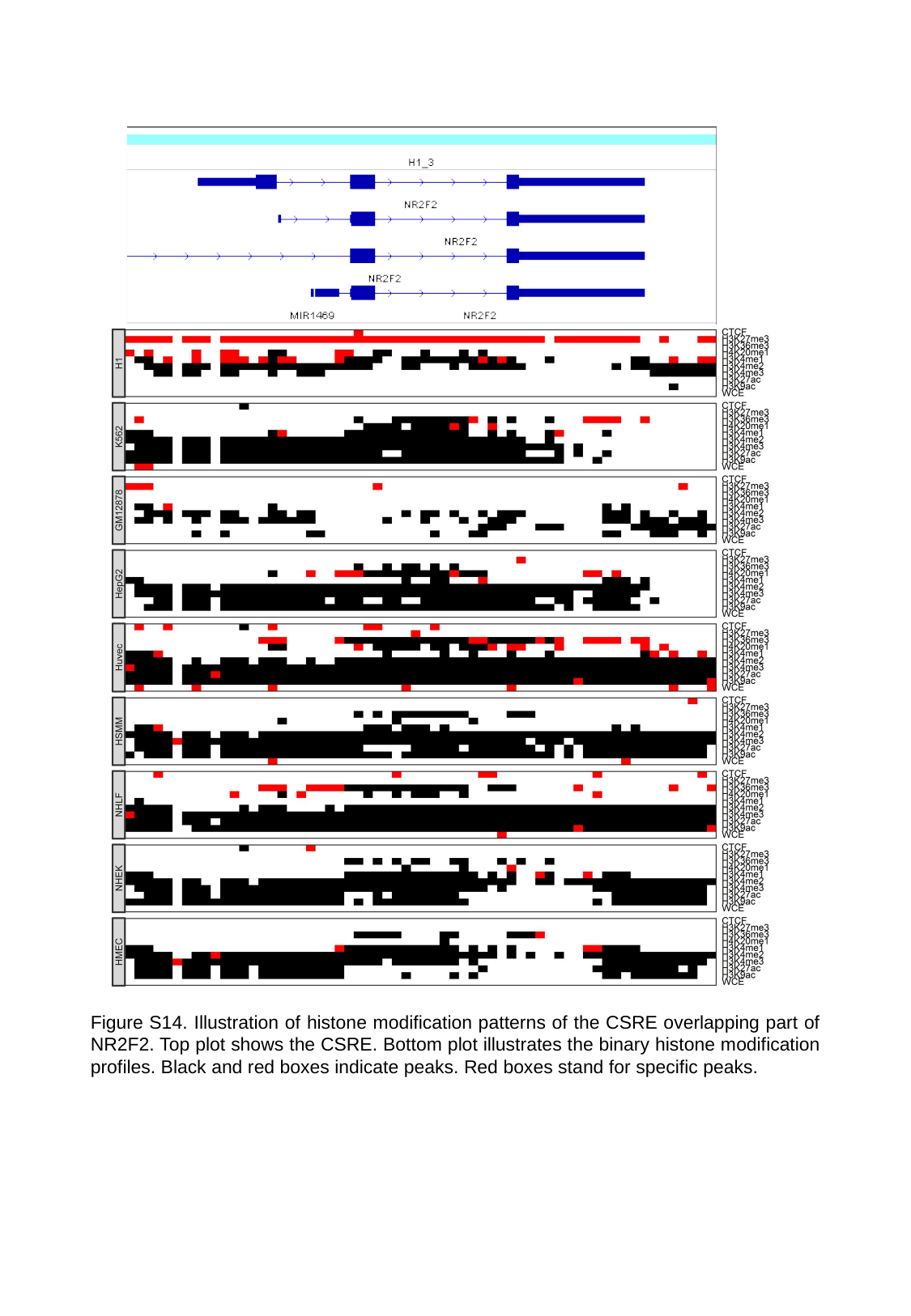

Figure S14. Illustration of histone modification patterns of the CSRE overlapping part of NR2F2. Top plot shows the CSRE. Bottom plot illustrates the binary histone modification profiles. Black and red boxes indicate peaks. Red boxes stand for specific peaks.

## Slide 16
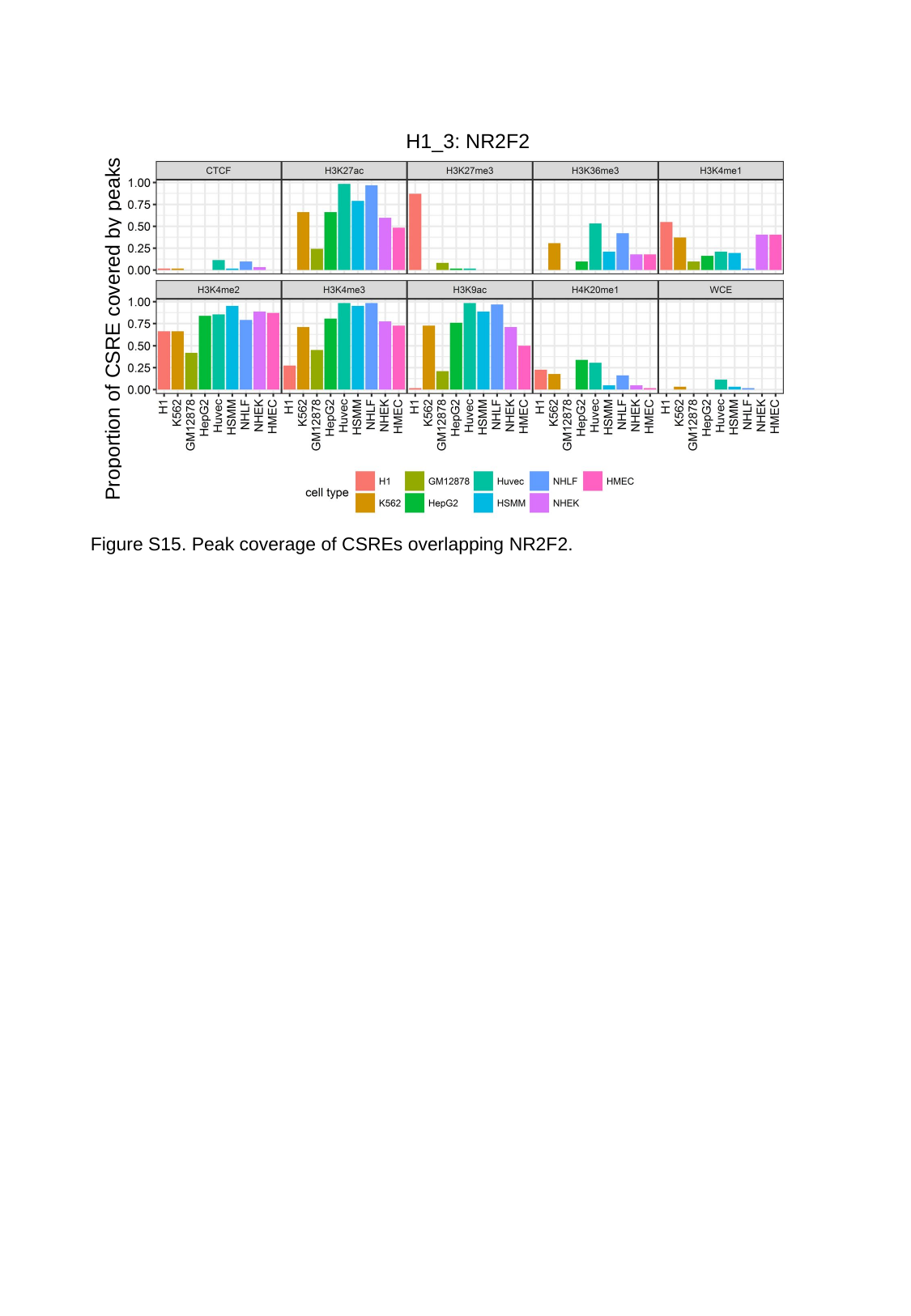

H1_3: NR2F2
Proportion of CSRE covered by peaks
Figure S15. Peak coverage of CSREs overlapping NR2F2.

## Slide 17
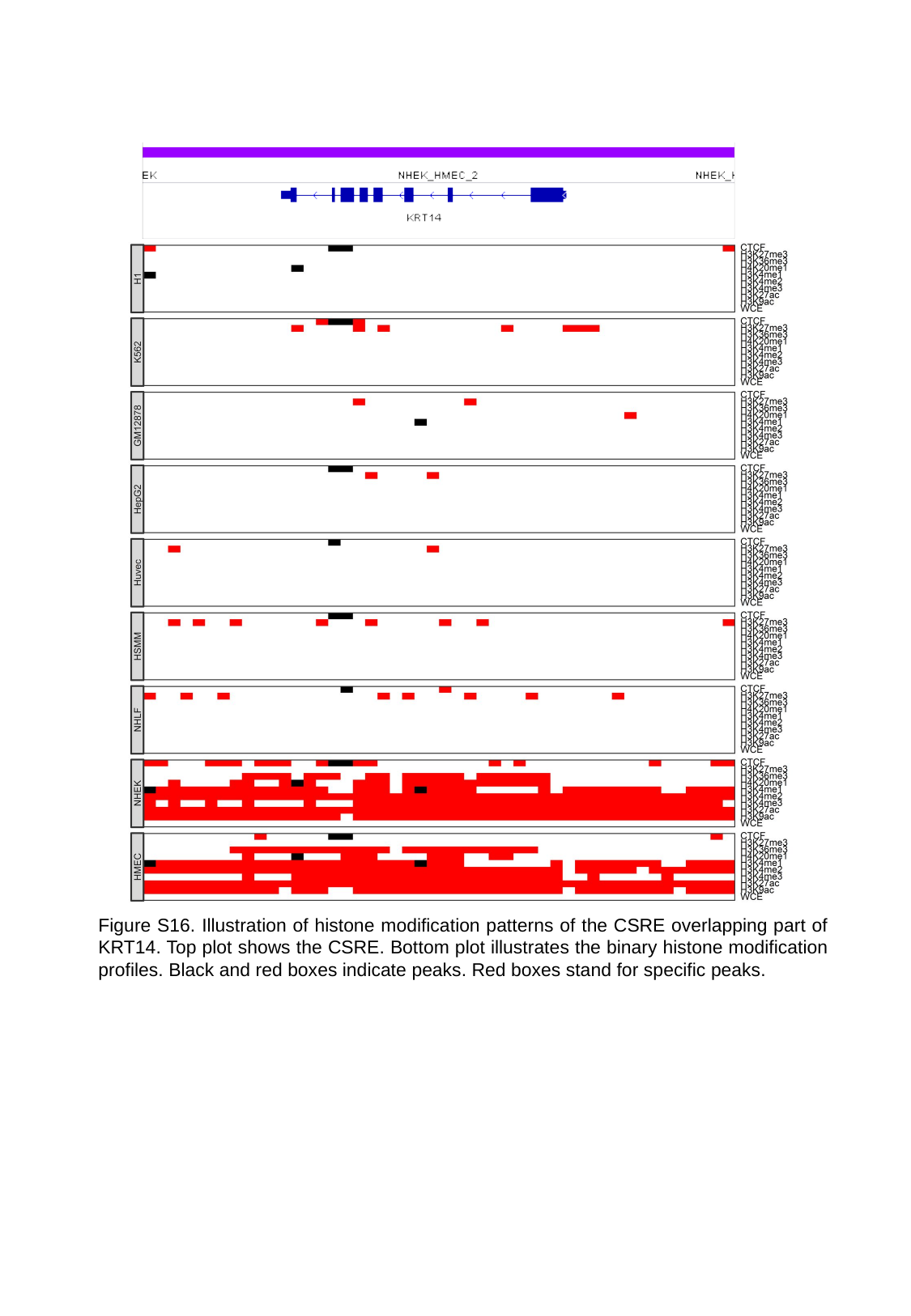

Figure S16. Illustration of histone modification patterns of the CSRE overlapping part of KRT14. Top plot shows the CSRE. Bottom plot illustrates the binary histone modification profiles. Black and red boxes indicate peaks. Red boxes stand for specific peaks.

## Slide 18
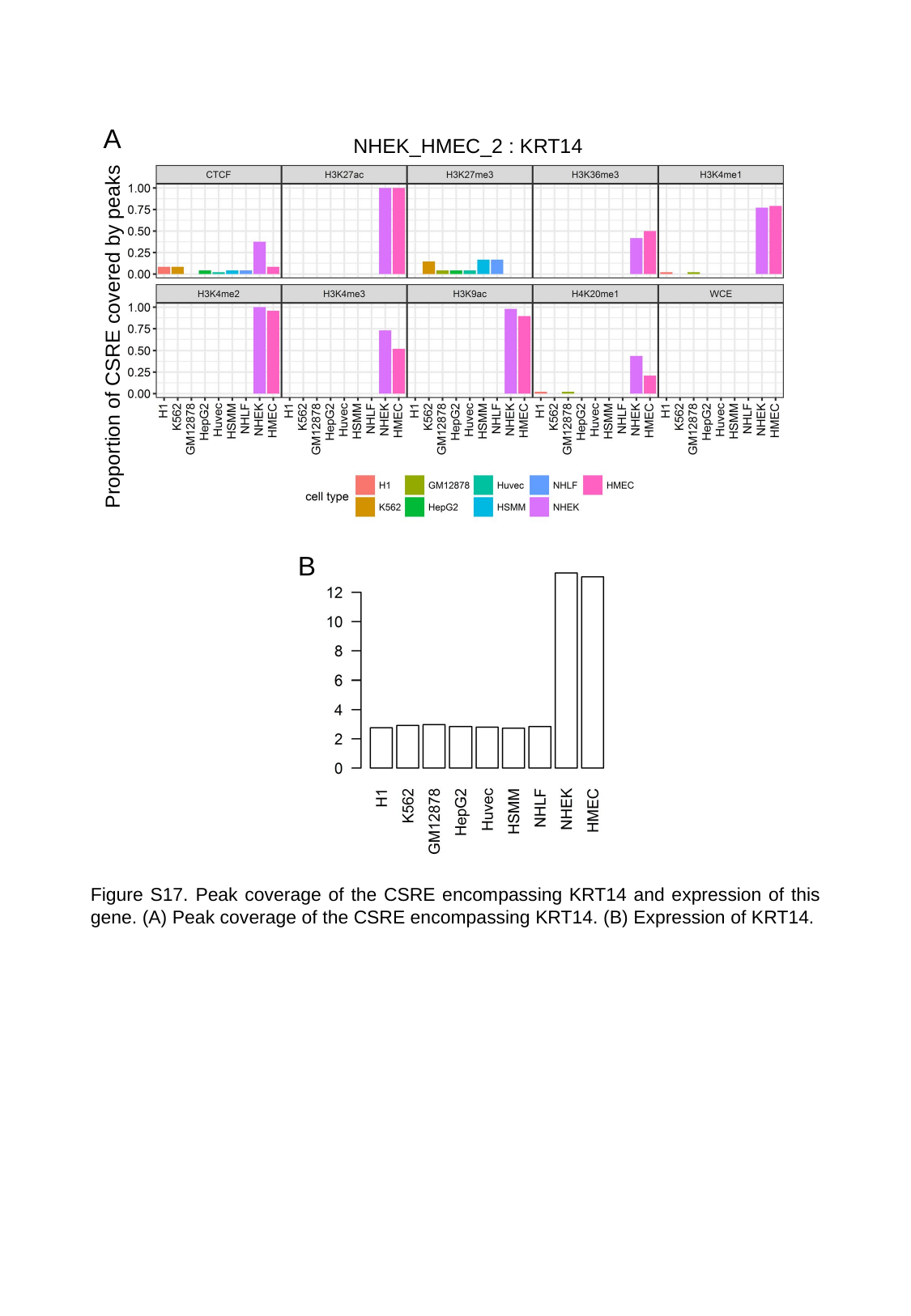

A
NHEK_HMEC_2 : KRT14
Proportion of CSRE covered by peaks
B
Figure S17. Peak coverage of the CSRE encompassing KRT14 and expression of this gene. (A) Peak coverage of the CSRE encompassing KRT14. (B) Expression of KRT14.
